# A review of the mechanisms that confer antibiotic resistance in pathotypes of *E. coli*

**DOI:** 10.3389/fcimb.2024.1387497

**Published:** 2024-04-04

**Authors:** Sina Nasrollahian, Jay P. Graham, Mehrdad Halaji

**Affiliations:** ^1^ Department of Bacteriology and Virology, School of Medicine, Shiraz University of Medical Sciences, Shiraz, Iran; ^2^ Environmental Health Sciences Division, School of Public Health, University of California, Berkeley, CA, United States; ^3^ Infectious Diseases and Tropical Medicine Research Center, Health Research Institute, Babol University of Medical Sciences, Babol, Iran; ^4^ Department of Medical Microbiology and Biotechnology, School of Medicine, Babol University of Medical Sciences, Babol, Iran

**Keywords:** *Escherichia coli*, antibiotic resistance, ESBL, efflux pump, UPEC

## Abstract

The dissemination of antibiotic resistance in *Escherichia coli* poses a significant threat to public health worldwide. This review provides a comprehensive update on the diverse mechanisms employed by *E. coli* in developing resistance to antibiotics. We primarily focus on pathotypes of *E. coli* (e.g., uropathogenic *E. coli*) and investigate the genetic determinants and molecular pathways that confer resistance, shedding light on both well-characterized and recently discovered mechanisms. The most prevalent mechanism continues to be the acquisition of resistance genes through horizontal gene transfer, facilitated by mobile genetic elements such as plasmids and transposons. We discuss the role of extended-spectrum *β*-lactamases (ESBLs) and carbapenemases in conferring resistance to *β*-lactam antibiotics, which remain vital in clinical practice. The review covers the key resistant mechanisms, including: 1) Efflux pumps and porin mutations that mediate resistance to a broad spectrum of antibiotics, including fluoroquinolones and aminoglycosides; 2) adaptive strategies employed by *E. coli*, including biofilm formation, persister cell formation, and the activation of stress response systems, to withstand antibiotic pressure; and 3) the role of regulatory systems in coordinating resistance mechanisms, providing insights into potential targets for therapeutic interventions. Understanding the intricate network of antibiotic resistance mechanisms in *E. coli* is crucial for the development of effective strategies to combat this growing public health crisis. By clarifying these mechanisms, we aim to pave the way for the design of innovative therapeutic approaches and the implementation of prudent antibiotic stewardship practices to preserve the efficacy of current antibiotics and ensure a sustainable future for healthcare.

## 1 Introduction

*Escherichia coli* is classified within the order Enterobacterales and family Enterobacteriaceae (1). *E. coli* exhibits notable versatility as a bacterial species, characterized by a comprehensive phylogenetic substructure. More than 160 *E. coli* serotypes have been categorized according to their major surface antigens, namely H (components of flagella), O (oligosaccharide polymer), and K (capsular polysaccharides) (Tuttle et al., 2021). *E. coli* can be classified into various groups based on its genetic substructures that are linked with different phylogenies. *E. coli* can be categorized using this method into the following groups: A, B1, B2, C, D, E, F, and clade. The pathogenic *E. coli* strains that cause infections outside the intestine often belong to groups B2 and D, while the non-pathogenic *E. coli* strains are typically classified as belonging to groups A or B1. Groups E and F are associated with the primary groups B2 and D. It is noteworthy that *E. coli* strains showing an identical phenotype but having different genetic makeup are categorized under the concealed clade I group. *E. coli* is a familiar commensal bacterium that colonizes the gut soon after birth. It competes successfully in the human gut and is the most prevalent facultative anaerobe in the human intestinal microbiota (Pais et al., 2022).

**Figure 1:**
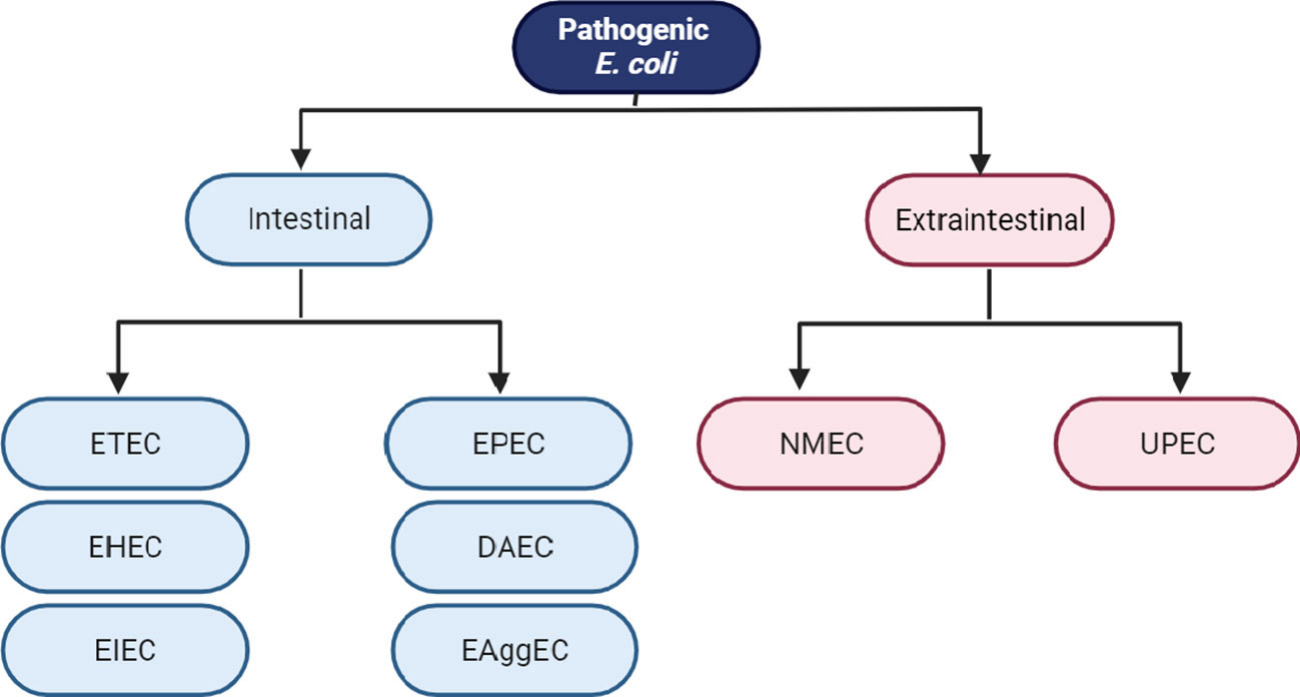
Classification schema of intestinal and extraintestinal pathogenic *E. coli* variants: Enterotoxigenic *E. coli* (ETEC), enteropathogenic *E. coli* (EPEC), enterohemorrhagic *E. coli* (EHEC), enteroinvasive *E. coli* (EIEC), enteroaggregative *E. coli* (EAEC), diffusely adherent *E. coli* (DAEC), neonatal meningitis *E. coli* (NMEC) and uropathogenic *E. coli* (UPEC).

Even though *E. coli* is a widely recognized bacteria that lives in harmony with its host, numerous hazardous strains of *E. coli* exist. Certain strains of *E. coli* have adapted to their environment and have developed particular virulence factors that enhance their ability to adjust to new surroundings, thereby enabling them to cause a range of illnesses, including both intestinal and extraintestinal infections. The classification of pathogenic *E. coli*: *E. coli* that produce Shiga toxins (STEC), enterohemorrhagic *E. coli* (EHEC), enteropathogenic *E. coli* (EPEC), enteroaggregative *E. coli* (EAEC), and diffusely adherent *E. coli* (DAEC), Shigella/enteroinvasive *E. coli* (EIEC), enterotoxigenic *E. coli* (ETEC) and adherent-invasive *E. coli* (AIEC). Diarrhea due to *E. coli* is classified particularly as a leading cause of mortality and desolation in nations that are still developing associated with bacterial infections among other pathogens. Infectious diarrhea is associated with moderate mortality and is generally less severe in industrialized countries but affects many people and represents a significant disease burden (**Figure 1**). These classifications are based on the acquisition of virulence factors and the characteristics of the resulting illness (Mafokwane et al., 2023; Mueller and Tainter, 2023).

The ailments comprise stomach flu, inflammation of the colon, bloody diarrhea, Hemolytic uremic syndrome (HUS), bladder infection, blood poisoning, lung inflammation, and brain membrane inflammation. Certain of these illnesses pose a considerable risk of death if not handled appropriately, hence it is crucial to combat them with highly potent antibiotics (Rahal et al., 2012). Nevertheless, in recent times, the primary worry has been the rising instances of foodborne epidemics in high income nations, brought about by harmful *E. coli* present in tainted meat, fruits, and vegetables (Mohamed and Habib, 2023). ExPEC is by far the leading cause of urinary tract infection (UTI) (hence the label uropathogenic *E. coli* [UPEC]) and bacteremia (Russo and Johnson, 2003). *E. coli* isolates are typically classified operationally as ExPEC based on their presumed intrinsic virulence potential as inferred from the presence/absence of specific putative or proven virulence genes irrespective of their immediate source of isolation. Thus, a molecular typing tool is needed for determination of whether an anonymous isolate is likely ExPEC or non-ExPEC. In contrast with ExPEC, the *E. coli* strains that cause diarrhea are referred to collectively as diarrheagenic *E. coli* (DEC) or intestinal pathogenic *E. coli* (IPEC). Additionally, in contrast with both ExPEC and DEC, nonpathogenic commensal *E. coli* strains colonize the human intestine without causing disease and may even be beneficial to the host by harvesting energy, protecting against other pathogens, or regulating host immunity. There is, however, a thin line between the definition of virulence and fitness factors in ExPEC and commensals. It has been suggested that ExPEC virulence might be a by-product of the commensal lifestyle (Malberg Tetzschner et al., 2020).

Simple UTIs, the most frequent bacterial infections worldwide, are caused by UPEC in between 50% and 90% of cases. Both genders can have UTIs, but women are more vulnerable due to their physiological and anatomical differences (i.e. the urethra is shorter and more proximal to the rectum). According to earlier studies, 50% of women and 15% of men, respectively, would get at least one UTI in their live s (Minardi et al., 2011; Whelan et al., 2023).

Managing UTIs is becoming increasingly difficult due to the growing resistance of UPEC to commonly prescribed antimicrobial drugs. Several recent guidelines have repositioned nitrofurantoin as a first-line antibiotic for the treatment of lower UTI. While the prevalence of nitrofurantoin resistance remained low (Giske, 2015). Besides nitrofurantoin, fosfomycin is another important oral antibiotic for the treatment of uncomplicated UTI. In several Asian countries, *fosA3* currently is the main mechanism of fosfomycin resistance in *E. coli* (Ho et al., 2013a, b). The commonly used antimicrobial agents for UTIs include *β*-lactams such as penicillin, cephalosporins, monobactams, and carbapenems (Raunak et al., 2022). In addition to the increasing frequency of *E. coli* infections, their persistent drug resistance is a serious issue. Enterobacteriaceae strains that are multidrug-resistant (MDR) extremely drug-resistant (XDR) and resistant to nearly all current antibiotics have emerged as a result of recent evolution (Kot, 2019). MDR are labelled as such because of their *in vitro* resistance to more than one antimicrobial agent. Infections with MDR can lead to inadequate or delayed antimicrobial therapy, and are associated with poorer patient outcomes. XDR means resistance to multiple antimicrobial agents, but also to their ominous likelihood of being resistant to all, or almost all, approved antimicrobial agents. From the Greek prefix ‘pan’, meaning ‘all’, pandrug resistant (PDR) means ‘resistant to all antimicrobial agents (Magiorakos et al., 2012). According to several studies, 4 of 19 low- and middle-income countries (LMICs) in Genes 2022 have high rates of MDR UPEC isolates, ranging from 42% in China to 49.8% in Iran and alarming 68% in Pakistan or even 98% in Mexico (**Table 1**). Age, recurrent UTIs, catheterization, previous use of antibiotics, hospitalization, nursing home stays, urinary tract abnormalities, and nursing home residence have all been identified as risk factors for the emergence of MDR strains (Genao and Buhr, 2012).

**Table 1:** The prevalence of MDR, XDR and PDR *E. coli* strains from recent studies across the globe.

Country	Year	Authors	Sample size	Clinical Source	MDR	XDR	PDR	References
**Iraq**	2023	Al-Hasani et al	113	Clinical sample	98.2%	21.24%	1.77%	(Al-Hasani et al., 2023)
**USA**	2023	Jennifer H Ku et al	233,974	Urine	12%	–	–	(Ku et al., 2023)
**Iraq**	2022	Abdulhameed et al	67	Clinical sample	46.3%	37.3%	16.45	(AbdulHameed and AbdulJabbar, 2022)
**Iran**	2022	Nasrollahian et al	76	Urine	66%	–	–	(Nasrollahian et al., 2022)
**Zambia**	2022	Bumbangi et al	1020		82.8%	3%	0.2%	(Bumbangi et al., 2022)
**Iran**	2021	Kalantari et al	139	Clinical sample	82.6%	48%	2.6%	(Kalantari et al., 2021)
**Iran**	2021	Zangane matin et al	406		90%	2.2%	1%	(Zangane Matin et al., 2021)
**Portugal**	2021	Olga Cardoso et al	340 *E. coli*	Stool	57.7%	3.5%	0.3%	(Cardoso et al., 2021)
**Sudan**	2020	Ehssan H. Moglad et al	69		49.3%	–	–	(Moglad, 2020)
**Brazil**	2019	Souza et al	681	Clinical sample	23%	–	–	(Souza et al., 2019)
**Nigeria**	2019	Abimiku, R. H et al	207	Stool	84%	7.2%	4.3%	(Abimiku et al., 2019)
**Bangladesh**	2019	Asma et al	1788	Urine	39.7%	5.4%	–	(Asma et al., 2019)

ESBL stands for Extended Spectrum *β*-Lactamase. *β*-lactamase enzymes break down and destroy some commonly used antibiotics, including penicillins and cephalosporins, and make these drugs ineffective for treating infections. ESBL production is associated with a bacterium usually found in the bowel. This resistance means that there are fewer antibiotic options available to treat ESBL-producing Enterobacterales infections. The primary curative antibiotics for ESBL-producing *E. coli* were cephalosporin and carbapenem; nevertheless, following the emergence of carbapenem-resistant *E. coli* isolates (CREC) and the global spread of these variants, polymyxin, tigecycline, fosfomycin, and aminoglycosides, either alone or in conjunction with other antibiotics, are the antibiotics that continue to be efficacious against CREC (Terbtothakun et al., 2021). Nevertheless, research has indicated that infections triggered by *E. coli* strains that are resistant to carbapenem are linked to elevated mortality rates as they are resistant to numerous categories of antibiotics, such as fluoroquinolones, trimethoprim-sulfamethoxazole, and broad-spectrum cephalosporins (Kot, 2019).

Various mechanisms of resistance are exhibited by *E. coli* strains, including the production of different *β*-lactamase enzymes, lowered permeability of the membrane, formation of capsule and biofilm, employment of efflux pumps, and enzymatic modification. *E. coli* strains that are MDR or XDR employ various means of dissemination, such as the transfer of high-risk mobile genetic elements – including plasmids and transposons – containing multiple antibiotic resistance genes (ARGs). Antibiotic resistance results in restricted options for therapy, extended hospitalization durations, elevated expenses for treatment, and increased mortality rates. The rise in resistance to antibiotics and the lack of effectiveness of antibiotic treatments highlight the pressing necessity for the creation of alternative methods for treating UTIs. Most studies indicate that antimicrobial therapy for UTI requires regular antibiotic susceptibility testing to minimize the spread of MDR isolates. However, in LMIC antibiotics can be purchased over the counter, many people will just go to a pharmacy rather than get a prescription. Additionally, ongoing analysis of the antimicrobial susceptibility profiles of isolates from the UPEC in various regions would be highly beneficial in preventing the establishment of MDR or XDR clones (Perez and Van Duin, 2013; Kot, 2019; Sarshar et al., 2020).

Furthermore, exploring the processes behind the origin and dissemination of novel instances of antibiotic resistance is crucial for refining the recommendations for empirical antibiotic therapy for UTIs. Therefore, the goal of this review is to assess recent research and updates on AMR and the mechanisms that underlie changes in antibiotic resistant UPEC strains. We will also look at the many strategies used by *E. coli* to build resistance to numerous antibiotics.

To find relevant documents describing the mechanisms for resistance in pathotypes of *E. coli*, we searched PubMed (https://pubmed.ncbi.nlm.nih.gov/), Scopus and Google Scholar using the following search terms: (“*Escherichia coli*” OR “uropathogenic *Escherichia coli*” OR “UPEC”) AND “ antibiotic resistance mechanisms “. We also searched the resulting reference lists to identify additional articles.

## 2 *E. coli* biofilms & antibiotic resistance

**Figure 2:**
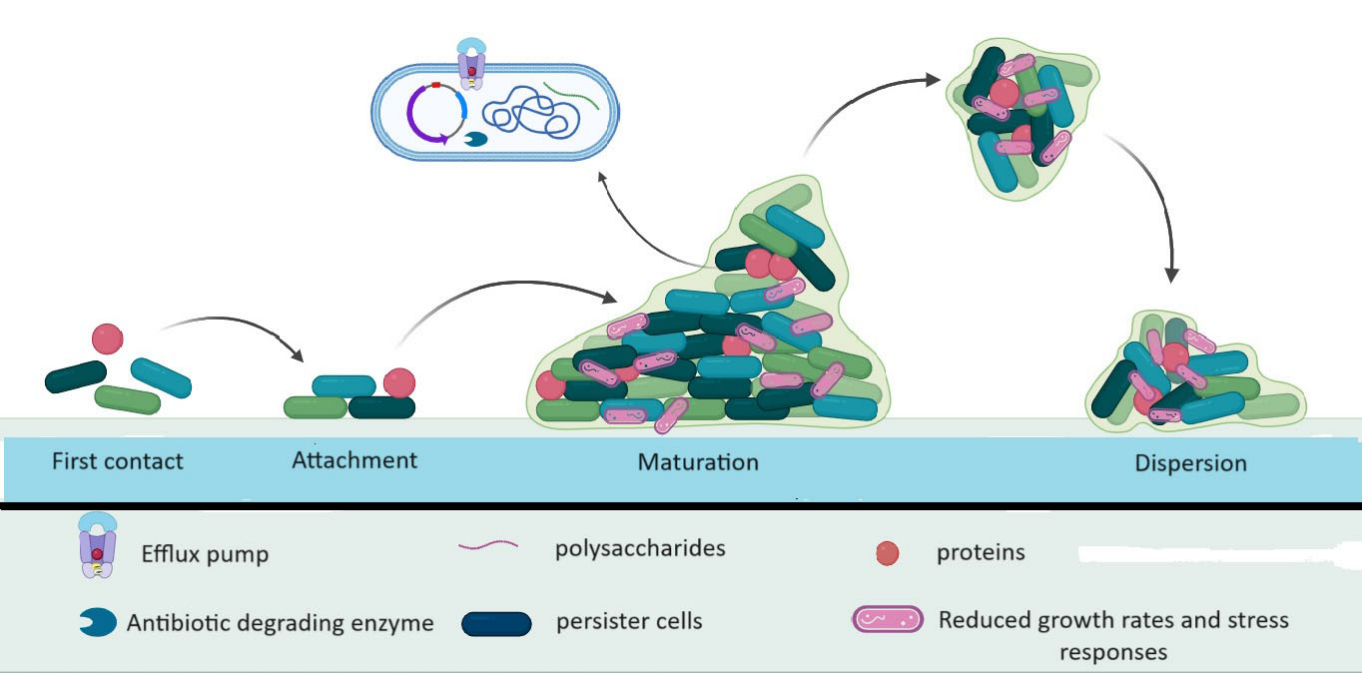
Schematic of Biofilm formation and mechanism of antimicrobial tolerance in biofilm.

Microorganisms use the formation of biofilms as a crucial survival strategy. Biofilm formation occurs in three main stages of biofilm formation have been identified: bacterial adhesion, quorum sensing (QS), and biofilm biomass formation (Li et al., 2023). Biofilms enable bacteria to withstand unfavorable conditions, including antibiotics. As a virulence factor, the biofilm enables the organism to adhere to both living and non-living surfaces and form tiny colonies that can multiply and protect themselves from the effects of antimicrobial treatments (Sharma et al., 2019; Nasrollahian et al., 2022). Apart from protecting pathogens against opsonization by antibodies, antimicrobial peptides, and phagocytosis, biofilms also prevent bacteria from being expelled by epithelial cells through the action of their cilia. Antibacterial chemicals are substantially less likely to harm bacterial populations in biofilms than they are to harm free-living bacteria. Because of this, biofilms can cause persistent infections that are difficult to treat, which is a serious public health concern (Nasrollahian et al., 2022). A self-made extracellular matrix that frequently consists of exopolysaccharides, amyloid fibers, secreted proteins, and extracellular DNA holds biofilms together and serves as their protective covering (Erskine et al., 2018). One process that facilitates the development of *E. coli* and helps it survive in the gastrointestinal, respiratory, and urinary tracts, ultimately leading to invasive infections (especially in individuals with weakened immune systems) and impeding its elimination is the creation of biofilms. Several genes, including *rpoS*, *sdiA*, and *rcsA* (biofilm-associated gene), play a significant role in biofilm formation and the acquisition of antibiotic resistance. Research has shown that the presence of adhesins such as FimH and MrkD is associated with the development of biofilms (Zhao et al., 2020) (**Figure 2**). Different chemical gradients being established throughout the biofilm community, in combination with limited diffusion and the metabolic activity of resident bacteria, leads to the emergence of distinct subpopulations that often cooperate metabolically but respond differently to the microenvironment depending on their location along the gradient (Penesyan et al., 2020). QS is a regulatory system that controls cell density which regulates gene expression by sensing the concentration of small-signal molecules called autoinducers (AIs) that are synthesized and released by bacterial cells. Specifically, inhibition of biofilm formation can be achieved by either interference with the QS mechanism, adhesion mechanism, or disruption of EPS. Genetically, each stage of biofilm formation of *E. coli* is associated with activating a different set of genes such as *luxS, fimH csgD*, and *bolA* that regulates the expression of its virulence factors. Particularly in *E. coli*, the *luxS* gene is required to produce AI-2, which mediates the QS mechanism, stimulates biofilm formation, and regulates the biofilm architecture (Alshammari et al., 2023).

In addition, the link between the development of biofilms and antibiotic resistance has drawn the interest of numerous researchers. Prior investigations conducted on *Pseudomonas aeruginosa* and *E. coli* have highlighted the significance of oxygen gradients in regulating the varied gene expression related to metabolic specialization and biofilm formation (Beebout et al., 2019). It has been observed in earlier studies that biofilm development can be stimulated by certain antibiotics when administered in low concentrations, implying that the control of biofilms may be a part of the overall reaction to external stressors, such as antibiotics (Penesyan et al., 2020).

*E. coli* is protected by biofilms from the immune system and antibiotic therapy. Compared to planktonic bacteria, bacteria in biofilms are up to 1000 times more resistant to drugs. The following processes are the primary causes of antibiotic tolerance among bacteria in biofilms: reduced growth rates and stress responses, limited antimicrobial penetration, persister cells, efflux pumps, and horizontal gene transfer (Ballén et al., 2022).

Currently, there are multiple records regarding the links between the resistance to antibiotics and the creation of biofilms by *E. coli* strains. *E. coli* employs various strategies to guard against different antimicrobial agents. The effects of various antibiotics on *E. coli* biofilm have been studied, including amikacin (AN), cephalosporins (CE), ceftazidime (CAZ), cefoperazone (CFP), cefepime (FEP), piperacillin (PEF), piperacillin-tazobactam (PTZ), ciprofloxacin (CP), gentamicin (GM), meropenem (MEM), and netilmicin (INN). These findings show that bacterial populations do not survive in their free-living state but do survive in biofilm. According to studies by Fei Zhao et al., *E. coli* exposed to ampicillin showed the highest rates of antibiotic resistance, followed by tetracycline, with resistance rates of 74.6% and 64.9%, respectively (Fuzi et al., 2017). As a result, some studies have shown that there is a significant association between the formation of biofilm and antibiotic resistance and virulence genes. Another study by Davari Abad et al. found a substantial relationship between the expression of the *sfa* (S fimbriae) gene and the development of robust biofilms (Davari Abad et al., 2019).

A study by Qian et al. explored the relationship between antibiotic resistance, biofilm formation, and biofilm-specific resistance in *E. coli* strains collected from children in China. According to the research, isolates that were susceptible to or had intermediate resistance to cefoxitin (CFX), ceftriaxone (CTX), cefazolin (CFZ), and GM were unable to produce as robust of biofilms as their resistant counterparts. This suggests that there is a correlation between the ability of a bacterium to form a biofilm and its resistance profile. Additionally, the results revealed that non-susceptible isolates exhibited higher levels of biofilm formation compared to susceptible isolates in six categories, namely quinolones, fluoroquinolones, cephalosporins, aminoglycosides, macrolides, tetracyclines, and nitrofurans. The formation of biofilm was found to be similar in *E. coli* strains that were susceptible and those that were not susceptible to *β*-lactams and lipopeptides (Qian et al., 2022). A systematic review and meta-analysis found that UPEC isolates that cause UTI frequently develop biofilms. The findings showed that out of 37 studies, 17 have looked into the relationship between the production of biofilms and antibiotic resistance. Of them, 14/17 (82.4%) showed a direct link between the development of biofilms and antibiotic resistance. Interestingly, ESBL producers and MDR isolates exhibited a significantly higher ability to produce biofilms (Garousi et al., 2022). According to recent research from Iran, 58% of the 107 UPECs that produced biofilm were ESBL producers, with 50% of them testing positive for the *AmpC* gene and 40% for the carbapenemase gene (Qasemi et al., 2022).

CRISPR/Cas systems in *E. coli* act as a defense mechanism against mobile genetic elements, such as plasmids and phages, which are involved in the horizontal spread of resistance genes. When *E. coli* encounters foreign genetic material, the CRISPR/Cas system can capture and integrate fragments of this material into its own genome. These captured sequences, known as spacers, serve as a memory of past infections. During subsequent encounters with the same genetic material, the CRISPR/Cas system uses these spacers to recognize and target the invading DNA for destruction. This defense mechanism helps *E. coli* combat the horizontal transfer of resistance genes carried by mobile genetic elements. By targeting and cleaving the DNA of these elements, CRISPR/Cas systems can prevent the spread of antibiotic resistance within bacterial populations. Studies have shown that CRISPR/Cas systems play a crucial role in limiting the acquisition of antibiotic resistance genes by bacteria like *E. coli*, thereby aiding in the control of antibiotic resistance development and spread (Mayorga-Ramos et al., 2023 and Dziuba et al., 2023).

Mobile genetic elements, such as plasmids and transposons, play a significant role in the horizontal transfer of resistance genes in *E. coli*. These elements can carry genes that confer resistance to antibiotics, heavy metals, and other environmental stresses. When these mobile genetic elements are transferred between bacteria, they can spread resistance traits rapidly within bacterial populations. In *E. coli*, the transfer of resistance genes via mobile genetic elements contributes to the development and dissemination of antibiotic resistance. This horizontal gene transfer allows bacteria to acquire new resistance mechanisms, leading to the emergence of multidrug-resistant strains that pose a serious threat to public health. The ability of mobile genetic elements to transfer resistance genes between bacteria highlights the importance of understanding and monitoring the spread of these elements in combating antibiotic resistance in *E. coli* and other pathogenic bacteria (Liu et al., 2023 and Jeon et al., 2023).

## 3 Production of different *β*-lactamases

The administration of four primary groups of *β*-lactam antibiotics, which comprise carbapenems, cephalosporins, penicillin, and monobactams, as novel therapeutic agents, has been associated with the proliferation of *β*-lactamases. The following section will examine ESBLs, carbapenemases, *β*-lactam/*β*-lactamase inhibitors, and AmpC *β*-lactamase.

### 3.1 β-lactam/β-lactamase inhibitor resistance

The treatment of infections brought on by Gram-positive and Gram-negative pathogens has relied heavily on the use of *β*-lactams, a large family of bactericidal medicines that prevent the manufacture of the bacterial cell wall. They may be grouped into four major categories: (i) cephalosporins, (ii) monobactams, (iii) penicillin derivatives, and (iv) carbapenems. The most frequently prescribed antibiotics for treating UTIs are the first two (amoxicillin or cefuroxime axetil), whereas the latter two are exclusively used to treat individuals with life-threatening illnesses (Rozwadowski and Gawel, 2022).

The widespread usage of *β*-lactams has resulted in the establishment and spread of resistance, much like with other antimicrobial groups. The target can be altered [through mutation or expression of alternative penicillin-binding protein (PBPs)], cell permeability can be decreased (by downregulating porins necessary for *β*-lactam entry), efflux systems can be overexpressed, and modifying or degradative enzymes can be produced (Tooke et al., 2019).

The prevalence of amoxicillin-resistant UPEC strains necessitates caution when treating UTIs with amoxicillin alone. *E. coli* isolates have been found to harbor numerous genes encoding resistance to *β*-lactam antibiotics, such as *bla*_TEM_, which is prevalent in *E. coli* and can degrade penicillin and first-generation cephalosporins, but only encodes small mRNA (Poirel et al., 2018).

A chromosomal *β*-lactamase that is encoded by some members of the Enterobacteriaceae family is active against a variety of *β*-lactam drugs (Smet et al., 2010). The plasmid-encoded extended-spectrum serine *β*-lactamase enzymes that were discovered in clinical strains hydrolyze all *β*-lactams aside from carbapenems. *β*-lactamase inhibitors, such as clavulanic acid, can prevent them from *β*-lactam antibiotics (Paterson and Bonomo, 2005).

To maintain the efficacy of *β*-lactams against *β*-lactamase-producing pathogens, the development of *β*-lactamase inhibitors has been crucial. However, these inhibitors are not typically developed as stand-alone medications due to their lack of antibacterial activity at clinically relevant levels. Instead, they are designed in combination with a partner *β*-lactam based on two key considerations: (i) the inhibitor’s ability to counteract *β*-lactamases that can hydrolyze the *β*-lactam, and (ii) similarity in pharmacokinetic properties to ensure that the *β*-lactam’s structural integrity is maintained over a specific dosing interval. Regulatory pathways for the development of these inhibitors as independent therapies are currently lacking (Yahav et al., 2020).

Clavulanic acid, sulbactam, and tazobactam are examples of *β*-lactamase inhibitors, which have a ring with the name *β*-lactam in their chemical structure. The effectiveness of sulbactam and tazobactam is wider compared to clavulanic acid as they can inhibit both class C cephalosporinases and class A *β*-lactamases including ESBLs. However, none of these inhibitors can effectively combat metallo-*β*-lactamases (MBL) and serine carbapenemases. To address this, other non *β*-lactam *β*-lactamase inhibitors like avibactam, vabrobactam, and relebactam have been developed (Bush and Bradford, 2020).

The *β*-lactam *β*-lactamase inhibitor combinations (BLBLIs), a significant class of novel antibiotics with broad-spectrum action, are a class of antibiotics in its own right. Clinical practice commonly uses combinations such as ampicillin/sulbactam (A/S), amoxicillin/clavulanic acid (A/C), piperacillin/tazobactam (PTZ), ceftazidime/avibactam (CZA), and ceftolozane/tazobactam (C/T) (Yahav et al., 2020) (**Table 2**).

## 4 Broad-spectrum *β*-lactam-*β*-lactamase inhibitor (BL-BLI) combinations

### 4.1 Ceftolozane-tazobactam

**Figure 3:**
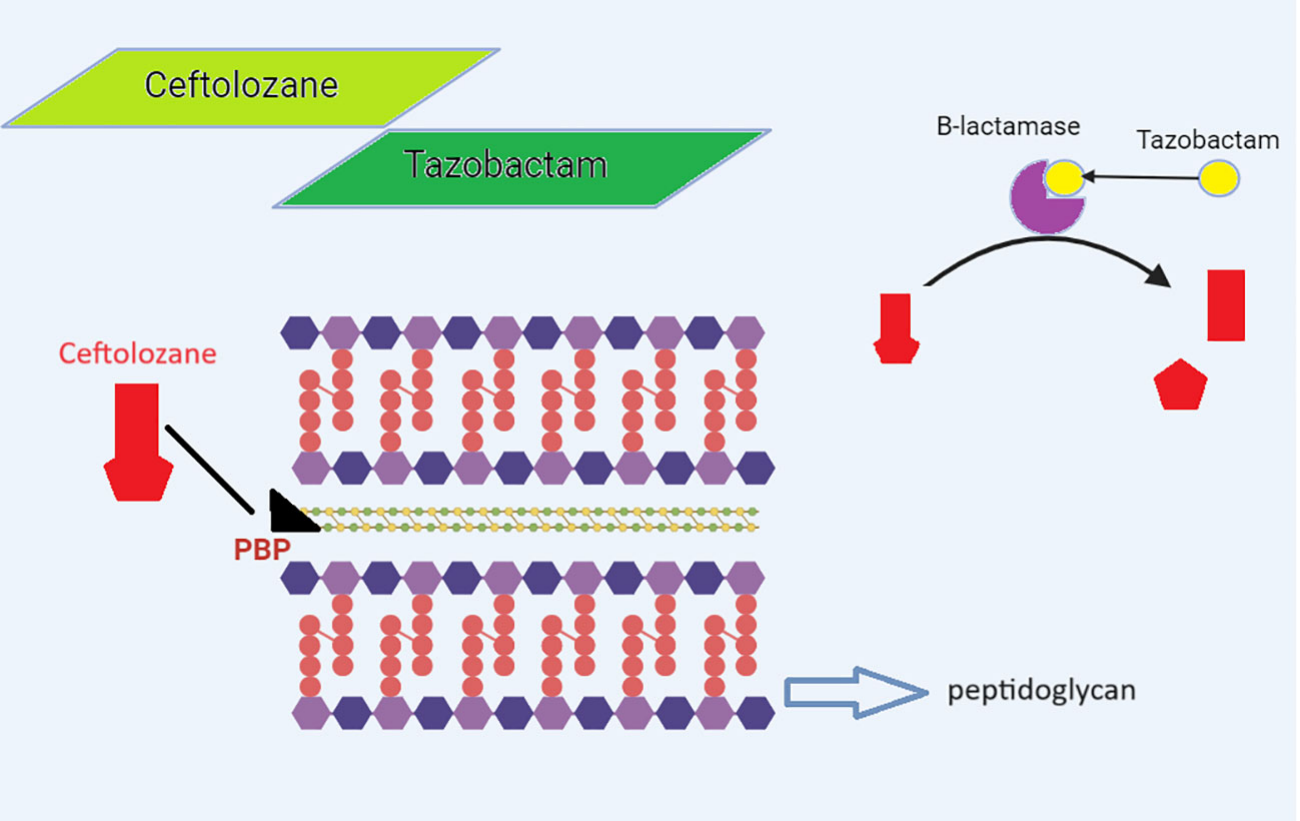
Mechanism of action of ceftolozane/tazobactam.

The management of carbapenem-resistant Gram-negative bacteria (GNB) has improved after the 2014 release of C/T, a new cephalosporin *β*-lactamase-inhibitor combination. A fifth-generation cephalosporin called ceftolozane and a bactericidal *β*-lactamase inhibitor called tazobactam make up the combination antibiotic known as C/T. Similar to other *β*-lactams, ceftolozane inhibits PBP (PBP1b, PBP1c, and PBP3), enhances outer membrane permeability, increases stability against efflux, and enhances stability against chromosomal AmpC *β*-lactamase, which prevents the production of bacterial cell walls and causes cell death (**Figure 3**) (Perovic et al., 2023). Ceftolozane can, however, be hydrolyzed by a few class A and B carbapenemases, including KPC-, VIM-, IMP-, and NDM-like enzymes; class D ESBLs, such as OXA-2 and OXA-10-like enzymes; and some class A ESBLs, like PER and GES, but not CTX-M-type enzymes. Tazobactam, which also blocks the hydrolysis of ceftolozane, inhibits the majority of class A *β*-lactamases and some AmpC cephalosporinases (plasmid-mediated class C *β*-lactamases). The drugs ceftolozane/tazobactam and C/A have been authorized in South Africa (SA) since 2022 (Mojica et al., 2022; Perovic et al., 2023).

C/T is licensed to treat complex intra-abdominal infections (cIAI), nosocomial pneumonia, and pyelonephritis in adults. In the United States, C/T has recently been licensed for the treatment of cUTI in pediatric patients (birth to 18 years old) (Gao et al., 2022). C/T was reported by Perovic et al. (2023) to be ineffective against the majority of MDR isolates found in South Africa (Perovic et al., 2023). In phase 2 Randomized Clinical Trial study (2023), C/T had a satisfactory safety profile that was comparable to meropenem and to the safety profile for C/T previously described in patients with cUTI. Additionally, C/T achieved outstanding clinical cure and microbiologic eradication rates. As a result, C/T is a unique, secure treatment choice for kids with cUTI that is effective against antimicrobial-resistant GNB, especially in newborns and young infants (Roilides et al., 2023). *E. coli* and *K. pneumoniae* isolates demonstrated greater sensitivity to C/T compared to PTZ in a thorough investigation conducted in Kuwait and Oman. To treat Enterobacterales that produce ESBL, it may be useful as a carbapenem-sparing antibiotic (Alfouzan et al., 2022).

Higher levels of *β*-lactamases, which could have surpassed the quantity of tazobactam that was accessible, may have contributed to some of the isolates’ reduced sensitivity to C/T. The existence of *β*-lactamase combinations, lesions in the OmpC-like and OmpF-like porins, and mutations in PBP3-ftsI might explain why Enterobacterales isolates are resistant to C/T (Karlowsky et al., 2022).

### 4.2 Piperacillin-tazobactam

A broad-spectrum *β*-lactam-*β*-lactamase inhibitor (BL-BLI) combination known as PTZ is widely used for the empirical treatment of serious infections such as bloodstream infections and infections linked to medical care. Some authors believe that PTZ might effectively cure severe infections caused by ESBL-producing GNB while reducing the need for carbapenems, which may promote the emergence and spread of carbapenemases. However, PTZ abuse has resulted in the emergence of resistant strains in recent years (Schuetz et al., 2018; Losito et al., 2022).

The surge in PTZ usage and the development of PTZ-resistant *E. coli* isolates in recent years have limited the therapeutic use of PTZ. The emergence of inhibitor-resistant TEM variants, the overproduction of TEM-1, or the development of TEM variants with improved hydrolytic capacity have all been implicated in the development of PTZ resistance in *E. coli*. The loss of porins, other *β*-lactamases such OXA-1 or AmpC, or both can result in PTZ resistance (Papp-Wallace, 2019).

An intriguing finding was made by Zhang et al. in 2023. 110 of the 195 patients who were a part of this trial received PTZ treatment, while 85 received meropenem. Between the PTZ and meropenem groups, the percentage of clinical cure was comparable (80% vs. 78.8%, p = 0.84). However, the PTZ group used antibiotics for a shorter amount of time overall, received effective antibiotic treatment for a shorter amount of time, and spent less time in the hospital. PTZ was safer than meropenem in the treatment of cUTIs in terms of side effects (Zhang et al., 2023). In a randomized controlled study, Seo et al. discovered that the effectiveness of cefepime in treating ESBL-producing UPEC-caused UTI was only 33.3% compared to PTZ (93%) and ertapenem (97%) (p 0.001) (Seo et al., 2017).

### 4.3 Ceftazidime-avibactam

Ceftazidime is hydrolyzed by class A ESBLs and carbapenemases, class B carbapenemases, and class C cepha-losporinases; most class D carbapenemases do not. Avibactam is a novel *β*-lactamase inhibitor of class A, class C, and some class D *β*-lactamases that has broad coverage of Gram-negative bacilli (Yahav et al., 2020).

CAZ-AVI, on the other hand, has little effect on isolates of Gram-positive bacteria, Gram-negative anaerobes, and class B *β*-lactamases. In published data, CAZ-AVI susceptibility has been documented for ESBL and AmpC-producing isolates. The combination was effective against isolates of *E. coli, K. pneumoniae*, *K. oxytoca*, and *Proteus mirabilis* that produced ESBL, AmpC, and CMY-like cephalosporinases. Additionally, the combination has demonstrated efficacy against bacteria containing the genes *bla_KPC-2_, bla_KPC-3_*, or *bla_OXA-48_* (Yahav et al., 2020; Guo et al., 2022).

Additionally, since NDM does not hydrolyze aztreonam, its activity is made possible by the inclusion of avibactam, which offers defense against class A enzymes. In their publication, a thorough analysis of *in vitro* experiments and clinical cases, Mauri et al. provided a comprehensive analysis of the effectiveness of the ATM-AVI combination in both laboratory and in-vivo against MBL-producing Enterobacterales. Their research suggests that the combination of ATM with either AVI or CZA is a viable treatment option for MBL-GN infections, with only a minority of isolates exhibiting high MIC values and the majority of treated patients experiencing favorable outcomes. Importantly, it has been shown that the *in vitro* antibacterial activity of the mixture ATM-AVI in Enterobacterales is unaffected by the presence of ceftazidime (Mauri et al., 2021).

In a multi-center investigation conducted in China in 2022, Sun et al, found that CAZ/AVI was active against nearly all KPC producers whereas ATM/AVI showed significant action against all CRE isolates, including MBL producers (Sun et al., 2022).

According to Taha et al. (2023), the combination of CAZ/AVI and ATM is regarded as a potent treatment approach, especially when used against *Klebsiella* spp. and *E. coli* isolates that produce several carbapenemase genes of metallo- and serine *β*-lactamases. This synergistic impact thus highlights the need for novel treatments for XDR and PDR-CPE (Taha et al., 2023). The effectiveness of combining CAZ/AVI and aztreonam against organisms that produce metallo-*β*-lactamases was also demonstrated by Sreenivasan et al. in 2022 (Sreenivasan et al., 2022). However, Ma et al, the use of aztreonam in conjunction with CAZ/AVI may be a viable therapy option because of the strong *in vitro* activity of ATM/AVI and the presence of ESBL-encoding genes (Ma et al., 2023).

CAZ/AVI showed outstanding *in vitro* efficacy against OXA-48, which produces CR-*E. coli* and CR-*K. pneumoniae*, in research conducted in India by Bakthavatchalam et al. (2022), and was equivalent to colistin and tigecycline in this regard. The results indicate that CAZ/AVI is a viable substitute for conventional therapy in the treatment of CRE infections with the OXA-48-like genotype (Bakthavatchalam et al., 2022).

Mecillinam combined with avibactam or CAZ/AVI significantly affects the majority of CPE types both *in vitro* and *in vivo*, according to research by Knudsen et al. The mecillinam/avibactam combination therapy may be a new, effective antibiotic treatment for MDR Gram-negative pathogens that produce carbapenemase (List et al., 2022).

The most common resistance mechanism to CAZ-AVI is class B and certain class D *β*-lactamases, such as OXA-24/40 in *A. baumannii*, but not OXA-10 or OXA-48 in Enterobacterales. Other factors contributing to resistance include heightened efflux pump activity, porin loss, and upregulation of the *bla_KPC_* gene. It should be noted that avibactam is unable to reverse resistance caused by single-point mutations in PBPs that lead to ceftazidime resistance (Yahav et al., 2020).

Numerous studies have noted the emergence of CAZ-AVI resistance following exposure, i.e., as a result of increased ceftazidime hydrolysis caused by mutations in the *β*-loop of *K. pneumoniae* carbapenemase (KPC) enzymes that are only partially inhibited by avibactam (Zhanel et al., 2018; Guo et al., 2022; Hernández-García et al., 2022).

CAZ/AVI resistance has reportedly been conferred by CMY mutations in recent years. According to Zhou et al., CMY-178 is a new CMY variation that mediates high-level resistance to ceftazidime-avibactam by improving ceftazidime hydrolysis and decreasing avibactam affinity. Notably, high-frequency horizontal transmission of *bla*_CMY-178_ was possible without incurring fitness costs (Zhou et al., 2023). In addition, a recent study by Hernández-García et al. found that strains of *E. coli* with the KPC-49 variant were resistant to ceftazidime/avibactam. These isolates can produce ESBLs as the high-risk clone ST131-H30R1-*E. coli*. The genes *bla-*_KPC-49_ were identified to be present on the transposon Tn4401a and were a component of the plasmid IncF (Hernández-García et al., 2021).

### 4.4 Cefepime-taniborbactam

Taniborbactam, which contains boronic acid, is a *β*-lactamase inhibitor that effectively inhibits AmpC, ESBL such as CTX-M and SHV and carbapenemases such as MBLs (VIM and NDM but not IMP) and serine-*β*-lactamases, such as OXA-48 and KPC-2. Therefore, it blocks class A, class B, class C and class D *β*-lactamases, which is a feature not presented by other b-lactamase inhibitors (Walker et al., 2022). As the medication forms a covalent bond with the serine residue at the enzyme-mediated hydrolysis site, serine *β*-lactamase is inhibited. The interaction between the boron moiety and the active zinc site, which results in a narrowing of the active site cleft, is what inhibits MBL. It has been proven that the combination of cefepime and taniborbactam provides significant efficacy against strains having a high MIC to CAZ/AVI. For the treatment of bacterial ventilator-associated pneumonia or hospital-acquired infections, cefepime/taniborbactam is being developed. Taniborbactam’s strong inhibitory effect was *in vitro* proven in NDM-1 and NDM-1 variations, according to Piccirilli et al. Cefepime/taniborbactam is an excellent alternative for treating severe infections because of its tremendous ability to perform against both ESBLs and MBLs (Piccirilli et al., 2021).

**Table 2:** The efficacy of different *β*-lactamase inhibitors belonging to the BLBLI class against various types of *β*-lactamase enzymes.

Antibiotics		Ceftazidime-avibactam, ceftolozane/tazobactam, meropenem/vaborbactam imipenem//relebactam
**Class A**	KPC	Avibactam, vaborbactam and relebactam are effective
	SHV	Avibactam, tazobactam, vaborbactam and relebactam are effective
	TEM	Avibactam, tazobactam, vaborbactam and relebactam are effective
	CTX-M	Avibactam, tazobactam, vaborbactam and relebactam are effective
**Class B**	MBL	All available BLI are ineffective
**Class C**	AmpC	Avibactam, vaborbactam and relebactam are effective
**Class D**	OXA	Avibactam is effective

BL/BLI resistance in *E. coli* advances from A/S to A/C and finally PTZ in a steady and unidirectional manner. The transition of low- or moderate-level resistance phenotypes (resistance to A/S with sensitivity to A/C and PTZ) into high-level BL/BLI resistance (resistance to A/S, A/C, and PTZ) is known as extended-spectrum BL/BLI resistance (ESRI), which we previously introduced (Galvez-Benitez et al., 2023).

### 4.5 Vaborbactam

Only a minority of the β-lactamases that vaborbactam, a non β-lactam, boronic acid-based β-lactamase inhibitor, shows activity against are ESBLs, KPC (Ambler class A), and AmpC-type enzymes (Ambler class C) (Bou Zerdan et al., 2022).

## 5 Extended Spectrum *β* -lactamases & carbapenemases

Particularly in Enterobacteriaceae, genes that produce ESBL and carbapenemase frequently have a high rate of transmission through plasmids that also carry other resistance genes. When both resistances are present in the same strain, they are often on different plasmids since ESBL and carbapenemases are connected with different plasmids (Tilahun et al., 2021).

Current research endeavors to elucidate the reasons behind certain lineages exhibiting greater adaptability in acquiring and retaining these plasmids while avoiding any negative impact on their fitness. It is suggested that these clones may be able to outcompete commensal strains of the same species and establish protracted intestine colonization through the appearance of adaptive mutations in intergenic areas and selective pressure on genes linked to anaerobic metabolism (Dunn et al., 2019).

As a result, it has been suggested that ESBL-producing bacteria may be resistant to antibiotics other than carbapenems, such as fluoroquinolones, tetracyclines, and sulfonamides. They were found to be either CTX-M enzyme variations or TEM and SHV variants. It is more common in environmental isolates, indicating that the environment contains ESBL-producing organisms. This has a significant impact on the use of antibiotics, the price of treatment, patient outcomes, and the range of available treatments (Rawat and Nair, 2010).

The use of carbapenem antibiotics is generally reserved for the treatment of MDR infections; nevertheless, the emergence of carbapenemases diminishes their therapeutic potency. Carbapenemases are *β*-lactamases that hydrolyze carbapenems and monobactams to variable degrees as well as penicillins, often cephalosporins, and other *β*-lactams (Kattan et al., 2008).

CRE has grown significantly in importance as a global public health issue since the initial report in the early 1990s. The extensive usage of ESBLs is believed to have increased the use of carbapenems and contributed to the emergence of carbapenem resistance. More than 13,000 hospitalized cases due to CPE occurred in 2017 alone, according to a study from the Centers for Disease Control and Prevention (CDC) (Goodman et al., 2019). Additionally, according to the Turkish Ministry of Health, in hospitalized patients in 2020, carbapenem resistance was found to be 20.1% in *E. coli* strains and 75.2% in *K. pneumoniae* strains (Uyanik et al., 2022).

According to recent investigations, CREs are found in water and cattle in addition to hospitalized people. Five important carbapenems are regarded as KPC, NDM, OXA-48-like, VIM, and IMP-type. ESBL-producing bacteria derived from clinical samples may be carbapenem-resistant and possess the genes *bla*_OXA-48_ *, bla*_NDM_ *_-1_, bla*_KPC_ *, bla*_IMP_, and *bla*_VIM_ (Uyanik et al., 2022).

Individual antibiotic resistance genes are associated with particular pathogenic clones that are molecularly categorized by their sequence type (ST) at the strain level. For instance, CREC is frequently linked to the ST167, ST617, ST410, and ST38 lineages of *E. coli*, while *E. coli* ST131 frequently expresses ESBL enzymes (Palomino et al., 2023).

Multiple plasmids carrying are associated with higher fitness costs for the host bacterium. The availability of carbapenemase-producing Enterobacteriaceae has limited the range of antibiotics that can be used to treat ESBL infections. As an alternative, a combination of *β*-lactam/*β*-lactamase inhibitors like PIP-TZM may be used to treat these infections. New studies indicate that the use of *β*-lactam/*β*-lactamase inhibitor antibiotics as an alternative to carbapenem antibiotics is a significant and advantageous strategy for empirical therapy. However, the increasing prevalence of *E. coli* strains resistant to *β*-lactam/*β*-lactamase inhibitors present a hurdle to this therapeutic approach (Wilson and Török, 2018).

### 5.1 Meropenem-vaborbactam

Meropenem/vaborbactam (MVB), a newly developed combination of carbapenem and *β*-lactamase inhibitor, has not been specifically investigated in the context of ST131, including its resistance-related H30R1 and H30Rx subclones (Johnston et al., 2021). MVB’s effectiveness against several *E. coli* isolates that produce *β*-lactamases were assessed by Amereh et al. The combination of meropenem and vaborbactam was most effective against KPC-producing bacteria, whereas it was ineffective against isolates that produced *β*-lactamases of the OXA-48 type. Our research was really useful in understanding how vaborbactam inhibits *β*-lactamase-producing bacteria (Amereh et al., 2022).

According to Johnston, MVB should be effective for treating *E. coli*-CRE infections that have spread internationally, essentially irrespective of other resistance traits, however this is likely to change depending on the local frequency of other *E. coli* lineages and carbapenem resistance mechanisms. Additionally, the majority of the MVB-resistant isolates in this investigation possessed genes for MBL or OXA-48 resistance (Johnston et al., 2021). Additionally, MVB had greater antibacterial activity (83% susceptibility) than other antibiotics in research from China, except colistin and tigecycline. MVB had noticeably strong activity against ST8 CREC isolates and KPC-producing isolates. Once the kind of carbapenemase, the susceptibility to MVB, and/or the susceptibility to STs are identified, it has a significant chance of being employed as a substitute for treating infections brought on by CREC (Huang et al., 2021). According to a randomized clinical trial conducted across multiple centers and countries, 98.4% of patients who received MVB as treatment were successful, compared to 94.0% of patients who were treated with PTZ, with a statistically significant difference (p = 0.001) (Kaye et al., 2018). Chang et al. reported that among the imipenem-intolerant isolates, 17 of 43 (39.5%) and 39 of 43 (90.7%) were each MVB and imipenem/relebactam (IMI-REL) sensitive. IMI-REL and MVB may be acceptable treatments for UTIs caused by Enterobacterales that are resistant to commonly recommended antibiotics (Chang et al., 2023).

### 5.2 Imipenem-relebactam

Except for manufacturers of metallo-carbapenemase, the combination of IMI-REL, approved by the FDA in 2019 and the EMA in 2020, has anti-carbapenem-resistant Enterobacteriaceae efficacy (Yang et al., 2022). For the treatment of complex UTIs, including pyelonephritis, and cIAIs brought on by sensitive GNB such *E. coli*, a well-established *β*-lactam and a novel *β*-lactamase inhibitor have been combined (Mansour et al., 2021). The antibiotic is also recommended for the management of ventilator-associated bacterial pneumonia (VABP) and pneumonia acquired in a hospital setting (HABP). According to the *in vitro* data, imipenem plus relebactam was substantially more effective than imipenem alone (Yang et al., 2022).

The serine *β*-lactamase inhibitor relebactam particularly inhibits class A (KPC, TEM, SHV, and CTX-M) and class C (AmpC, CMY) enzymes (Ball et al., 2016). Relebactam has minimal efficacy against class D enzymes that resemble OXA-48 but does not block the activity of class B enzymes such NDM-type carbapenemases (Bouvier et al., 2023). In a prior investigation, 200 isolates of Enterobacteriaceae that produce carbapenemase and were collected in the US between 2013 and 2017 had imipenem MIC values ranging from 2 to >16 µg/ml. Relebactam lowered the range of MIC values to 0.125-4 µg/ml as compared to imipenem monotherapy, with lower MIC_50_ (from 8 to 0.125 µg/ml) and MIC_90_ (from >16 to 0.5 µg/ml) values (Carpenter et al., 2019).

Clinical data investigations showed that the combination of IMI-REL and the treatment of imipenem-nonsusceptible infections was linked with favorable clinical response and safety in patients (Gaibani et al., 2022). Specifically, the RESTORE IMI-1 clinical trial established the comparability in terms of efficacy and tolerability between IMI-REL and the combination of imipenem and colistin for treating infections induced by imipenem-resistant pathogens. Furthermore, when compared to PTZ, the RESTORE IMI-2 study validated the effectiveness and safety of IMI-REL in the treatment of hospital-acquired bacterial pneumonia and ventilator-associated bacterial pneumonia (HABP/VABP) (Brown et al., 2020).

As of now, a limited number of Enterobacterales strains that produce carbapenemases and exhibit resistance to imipenem-relebactam have been detected. Among various mechanisms, it is primarily class B and D carbapenemases that play a significant role in conferring resistance to IMI-REL in CRE. IMI-REL resistance is frequently seen in organisms that produce these carbapenemases, as was previously noted (Karlowsky et al., 2018; Lob et al., 2020). Numerous investigations showed that a variety of mechanisms, such as mutations in carbapenemases, increased carbapenemase expression, mutations leading to changes in PBPs expression or function, heightened efflux activity, and reduced membrane permeability could also potentially play a role in the development of resistance to IMI-REL (Gaibani et al., 2022).

### 5.3 Plasmid-mediated AmpC- β -lactamase

In *E. coli*, class C *β*-lactamases known as AmpC-type enzymes acquire severe resistance to cephalosporins (Tamma et al., 2019). Many GNB have abundant chromosomes that contain AmpC-type enzymes. The *E. coli* enzyme has a special place in the history of *β*-lactamase research being the first *β*-lactamase to be discovered (Poirel et al., 2018).

The AmpC *β*-lactamase-encoding gene in *E. coli* can either be on the chromosome (cAmpC) or be connected with a plasmid (pAmpC). Because it is regulated by a strong attenuator and a weak promoter, cAmpC is produced at low levels. Many of the most significant opportunistic Gram-negative infections possess chromosomal genes for class C enzymes that are normally not expressed, generally designated as AmpC. However, derepression of these can result in high-level expression and an increase in MICs for susceptible *β*-lactams, either as a result of mutation or through induction by particular *β*-lactams (Cole et al., 2022).

Penicillins, the majority of cephalosporins, cephamycins, and monobactams are all hydrolyzed by cAmpC when it is expressed constitutively, whereas carbapenems and the fourth-generation cephalosporins are not. Additionally, “classical” *β*-lactamase inhibitors do not block AmpC *β*-lactamases (Longhi et al., 2022).

In 1989, *K. pneumoniae* strains obtained from South Korea harbored the initial plasmid-encoded AmpC variant. This variant was labeled as CMY-1 due to its association with a distinctive phenotypic feature linked to cephamycinase and its established resistance to cefoxitin (Aryal et al., 2020; Bush and Bradford, 2020).

Additionally, Pérez-Pérez and Hanson identified six families of AmpC *β*-lactamases as EBC, MOX, FOX, CIT, DHA, and ACC, among which CMY-2 of the CIT-type has the largest incidence globally (Pérez-Pérez and Hanson, 2002; Philippon et al., 2002). One of the most prevalent genes in a previously studied group of Polish UPEC isolates was recognized to be the *bla*_CMY-2_ (Adamus-Białek et al., 2018). In clinical isolates, *bla*_CMY-2_ gene and IncI1 plasmids were shown to be highly associated (Poirel et al., 2018). Therefore, the identification of strains that are AmpC-positive is crucial for providing the right care. According to the examined data, Asia, Oceania, and the Middle East have a higher prevalence of pAmpC-BL than the rest of the world. Europe has been reported to have the lowest prevalence, followed by America (Rodríguez-Guerrero et al., 2022).

According to Sadeghi, the MOX, EBC, and CIT genes were present in 7 (14.6%), 4 (8.3%), and 9 (18.8%) of the cases, respectively. In none of the samples were the genes for DHA, FOX, or ACC found (Sadeghi et al., 2022). The results of this study’s multiplex PCR experiment revealed that 1 isolate (2.1%) had 3 kinds of pAmpC cluster genes (*bla_MOX_* + *bla_EBC_* + *bla_CIT_*) present. Additionally, it was discovered that the frequency of the *bla*_CIT_ *, bla*_MOX_, and *bla*_EBC_ genes in *E. coli* isolates was 10.4, 8.3%, and 4.2%, respectively (Jomehzadeh et al., 2021).

The urogenital tract of elderly women appears to be the pathogen’s primary target, according to a surveillance study done in Canada on the organism that produces CMY-2. Patients receiving cephalosporin-based medication may not receive the best clinical care if strains that overproduce AmpC are not identified. Additionally, it has been suggested that porin mutations and AmpC overproduction may lessen the sensitivity to carbapenems (Longhi et al., 2022).

In undeveloped nations, prevalence rates range from 5% to 44.3%. Penicillins, cephems, and monobactams are no longer effective against bacteria that have been exposed to AmpC *β*-lactamases, although carbapenems continue to be effective. In these circumstances, adding *β*-lactamase inhibitors like clavulanate, sulbactam, or tazobactam is ineffective at combating antibiotic resistance (Muriuki et al., 2022).

IMI-REL proved effective against Enterobacterales with impermeability characteristics (such as the lack of porins) in research by Livermore et al. The action of imipenem is increased by relebactam’s inhibition of AmpC (Livermore et al., 2018; Yahav et al., 2020).

Temocillin is recommended as an intravenous treatment for severe UTIs and bloodstream infections, demonstrating efficacy against both ESBL and AmpC production (70). Delroy et al. conducted a retrospective observational study on adults with a UTI brought on by ESBL-producing UPEC. They discovered that 94% of the 72 patients treated with temocillin and 99% of the 72 patients treated with a carbapenem achieved clinical remission (p = 0.206). As a result, temocillin may be an option for treating UTI caused by ESBL and AmpC-producing UPEC. Furthermore, neither ESBLs nor AmpC *β*-lactamases had an impact on tigecycline (Delory et al., 2021).

## 6 Enzymatic modification mechanisms

Some molecular tactics for antibiotic resistance are used by bacteria, including mutation, post-translational modification, and avoidance of antibiotic binding. Antibiotics may also be broken down inside the cell if they are unable to alter the bacterial metabolism. In this process, the participation of enzymes is crucial (Munita and Arias, 2016).

### 6.1 Enzymatic modifications for protein synthesis

The most varied group of antibiotics, those that target the ribosome, are classified into eight different chemical groups. These antibiotics interfere with distinct ribosomal areas that they target and even overlap with, preventing certain stages of protein translation. Drugs made from aminoglycosides work to inhibit one or more translation-related biochemical processes to target the ribosome. The aminoglycoside family of antibiotics is another significant group of drugs. They are amino sugars that are connected to an aminocyclitol, a hexagonal cyclic alcohol, through glycosidic linkages (Lin et al., 2018).

*E. coli* has developed several strategies for resisting aminoglycosides, including efflux pumps, target site alterations, and enzymatic inactivation by aminoglycoside-modifying enzymes (AMEs) The primary mechanism of resistance is aminoglycoside inactivation by AMEs, based on the frequency and severity of resistance (Garneau-Tsodikova and Labby, 2016).

At present, UPEC isolates have revealed the existence of three distinct classes of AMEs: aminoglycoside nucleotidyltransferases or adenylyltransferases (ANTs), aminoglycoside phosphotransferases (APHs), and aminoglycoside acetyltransferases (AACs) (Soleimani et al., 2014; Azimi et al., 2022). By catalyzing changes at certain amino or hydroxyl groups, these enzymes reduce the drug’s capacity to bind to the ribosome (Lin et al., 2018). The two most frequent acetyltransferases identified in *E. coli* strains are AAC (3)-II/IV and AAC (6)-Ib. The two most prevalent nucleotidyl transferases in *E. coli* strains, ANT (2′′) and ANT (3′′, encoded by the *aadB* and *aadA* genes, respectively, and are frequently linked to integrons (Urban-Chmiel et al., 2022). The APH (6)-Ia and APH (6)-Id, which are encoded by the *strA* and *strB* genes, respectively, are present in all strains of *E. coli* (Guo et al., 2022).

Aminoglycosides and macrolide antibiotics are among the protein synthesis inhibitors that are resistant to RNA modification enzymes such as rRNA methyltransferase. Both Gram-positive and GNB infections are frequently treated with these antibiotics in clinical settings. They accomplish this by adding a methyl group to a few specific nucleotides of the 16S or 23S rRNA gene (Egorov et al., 2018). In GNB strains, several genes (*aviR, cfr, emtA, ermA, ermB*, and *ermC*) carry the genetic information for 23S rRNA methyltransferases, which confer resistance to antibiotics like lincosamides, phenicols, oxazolidinones, pleuromutilins, and streptogramin-A (Long et al., 2006; Das and Bhadra, 2020). Kasugamycin resistance, however, results from *E. coli* lacking Ksg methyltransferase. Similar to this, the deletion of the pseudouridine synthase *rulC* gene, which changes the 23S rRNA, results in considerable resistance among several enteric pathogens to clindamycin, linezolid, and tiamulin (Das and Bhadra, 2020).

Antibiotic resistance can be obtained by methylating the 23S rRNA; the type of resistance that results depends on where the methylation sites are located. Because their binding sites significantly overlap, a single methylation event can often confer resistance to several types of antibiotics. N-methylation, a mechanism linked to a well-known family of Erm N-methyltransferases, is one of the most prevalent changes observed in 23S rRNA. It methylates the N6 position of A2058, which is situated on the produced peptide’s active site (Dzyubak and Yap, 2016). Erm N-methyl transferase methylation of A2058 leads to resistance to lincosamide, macrolide, and streptogramin B, but not to resistance to ketolides, which are a subclass of macrolides.

Studies using a genomic approach, such as those by da Silva et al. (2019) in Brazil, showed that UPEC strains contain the aminoglycoside adenylases *aadA1*, *aadA2*, and *aadA5*, as well as the aminoglycoside phosphotransferases *strA*, *strB*, and aac(3)-Iva. The majority of the time, extrachromosomal entities like plasmids, integrons, and transposons contain these resistance-determining genes (da Silva et al., 2019).

In a Polish investigation, 78% of UPEC strains tested positive for *aac(3)-II (* Adamus-Białek et al., 2018 *)*. *Aac(3)- IV* was present in 25.7% of the UPEC isolates analyzed by Mashayekhi et al. in Iran (Mashayekhi et al., 2014). In China, 85% of UPEC isolates from female patients were *aac(3)-IIa*-positive for aminoglycoside resistance (Zeng et al., 2021).

### 6.2 Enzymatic modification for DNA replication

DNA gyrase and topoisomerase IV (topo IV) are two crucial type II enzymes present in *E. coli.* These two enzymes are extremely important for DNA replication. Topo IV, an important enzyme from *E. coli*, unlinks the daughter chromosomes for correct segregation during cell division (Levine et al., 1998). By site-directed interaction with specific amino acids of enzymes encoded by *gyrA, gyrB, parC*, and *parE*, which are their primary and secondary targets, quinolones and fluoroquinolones inhibit DNA topoisomerases II and IV. Thus name this particular interaction site as the quinolone resistance-determining region (QRDR) (Hooper and Jacoby, 2016).

Furthermore, it has been demonstrated that genes for quinolone resistance mediated by plasmids (PMQR) reduce fluoroquinolone susceptibility. One of these genes, the target protection PMQR gene *qnr* (*qnrA, qnrB, qnrC, qnrD*, and *qnrS*), generates proteins that prevent damage to the quinolone targets gyrase and topo IV (Strahilevitz et al., 2009; Jacoby et al., 2014).

The first chromosomal *qnr* gene, *mfpA*, was discovered in *Mycobacterium smegmatis* by Montero et al (Montero et al., 2001). MfpA homologs have so far been found in a wide variety of species, including UPEC (*mcbG* gene). The pentapeptide repeat protein family includes Qnr proteins (Rozwadowski and Gawel, 2022). Furthermore, PMQR is associated with the efflux pump genes *qepA* and *oqxAB*.

The MIC values for certain hydrophilic quinolones such as norfloxacin, ciprofloxacin, and enrofloxacin were found to increase significantly by a factor of 8 to 32 in the presence of QepA (Rahman et al., 2017). On the other hand, OqxAB was found to have a wider range of substrate specificity which included quinolones (ciprofloxacin, norfloxacin, and nalidixic acid), trimethoprim, and chloramphenicol (El-Badawy et al., 2017). Numerous investigations have been made to determine if isolates from people and food animals contain PMQR (Shaheen et al., 2013; El-Badawy et al., 2017; Ayobola et al., 2021; Lee et al., 2021). However, few research discuss the presence and the function of PMQR determinants and mutations in inducing resistance in Enterobacteriaceae isolates from mammalians, such as those discovered in *gyrA*, *parC*, and *parE* (Strahilevitz et al., 2009; Piekarska et al., 2022; Shariati et al., 2022). Recent research conducted by Azargun et al. identified the primary alterations associated with fluoroquinolone resistance in *E. coli*, which include substitutions at Ser80Ile and Glu84Val within the ParC subunit, along with Ser83Leu and Asp87Asn substitutions in the GyrA subunit (Azargun et al., 2019). The research conducted by Ostrer et al. supports these observations. They found that, among over 10,099 *E. coli* genomes examined, mutations at ParC84, GyrA83, or ParC80, GyrA87 were the most commonly encountered variations associated with fluoroquinolone resistance. These mutations in the GyrA and ParC subunits are situated within the quinolone resistance-determining region (QRDR), specifically close to the active site tyrosines (Tyr122 for GyrA and Tyr120 for ParC) of these enzymes (Ostrer et al., 2019). Prominent and widely distributed lineages of *E. coli*, like ST131H30, exhibit dual mutations in the specified sites within the *gyrA* and *parC* genes. These mutations not only indicate a high degree of resistance but also seem to provide a cost-effective advantage for the host bacterium (100). Minor clones, in contrast to widespread clones, primarily carry single mutations as opposed to double mutations. Instead, they produce efflux pumps to tolerate exposure to fluoroquinolones. This finding explains why *E. coli* consecutive clones favor energy-consuming mechanisms like efflux pumps over cheaper ones like double mutations in *parC* or *gyrA* with greater effectiveness. Alterations in DNA repair genes can contribute to antibiotic resistance in *E. coli*, with the exception of DNA replication gene alterations, which are mostly important to fluoroquinolone resistance (Ostrer et al., 2019). The S83L alteration was found in 98.9% of the QR isolates, with 34 isolates harboring S83L alone and 52 isolates harboring both S83L and D87N substitutions, according to Mirzaii et al (Mirzaii et al., 2018). Numerous additional investigations have discovered the S83L and D87N alterations in QR *E. coli* isolates, supporting the idea that these locations are crucial for drug binding and are likely to evolve as resistance emerges (Marcusson et al., 2009; Johnning et al., 2015; Aworh et al., 2023). S80I substitution was also discovered to be the most prevalent mutation in ParC in the examined QR *E. coli* isolates in a research conducted in Brazil by Minarini et al (Minarini and Darini, 2012). However, the E84V mutation was not found by the same scientists, who instead discovered E84G, E84A, and E84K replacements at this location of ParC. The most prevalent PMQR gene in *E. coli* isolates was *qnrS*. Numerous investigations from different countries have discovered that 1.5–14% of *E. coli* isolates include *qnrS* (Zhao et al., 2015; Rezazadeh et al., 2016; Aabed et al., 2021; Silva et al., 2023). Similar to earlier investigations, only 2.3% of QR *E. coli* isolates in the Mirzaii study had both *qnrA* and *qnrB* detected (Mirzaii et al., 2018). The *aac(6′)-Ib-cr*, *qepA*, and *oqxAB* genes, which are additional plasmid-mediated quinolone resistance, were found in *E. coli* strains. Tetracycline resistance is primarily mediated by the *tet(A)* gene in all *E. coli* strains that possess the *aac(6′)-Ib-cr* gene (Ruiz et al., 2012). This gene, *aac(6′)-Ib-cr*, encodes an aminoglycoside acetyltransferase that results in reduced susceptibility to ciprofloxacin.

Additionally, the co-existence of ESBL protein-encoding genes in PMQR strains leads to the development and selection of MDR-UPEC (Paniagua-Contreras et al., 2018; Sadeghi et al., 2020). In the study by Basu et al., co-resistance to *β*-lactams and quinolones was also examined. A first-line antibiotic used in UPEC therapy, ciprofloxacin, was only partially effective against 86% of the MDR strains that were identified from UTI patients. The majority of quinolone-resistant bacteria (50%) had PMQR, and every isolate also had at least one gene producing a *β*-lactamase, with *bla_TEM_* being the most prevalent. This work also shows that, with ciprofloxacin selection, resistance genes may be transmitted through conjugation (Basu and Mukherjee, 2018).

## 7 Efflux pumps

All bacteria possess efflux pumps, which are the main mechanism for MDR and drug resistance. Bacterial genomes often encode a number of efflux pumps, which are in charge of removing a wide range of substances from inside the cell. When overexpressed, efflux pumps have a significant impact on both the intrinsic drug sensitivity of a bacterial species as well as the development of clinically significant antibiotic resistance. The increased expression of efflux pump genes could be due to mutations in regulators or the insertion of IS elements that act as powerful promoters upstream of these genes. Mobile genetic elements can also be used to acquire new pump genes (Li and Nikaido, 2009; Soto, 2013).

Efflux pumps, which facilitate bacterial pathogenicity and medication resistance, are attracting medical professionals seeking treatments for MDR, with drug efflux pump inhibition potentially treating various health issues. Tetracycline-specific efflux pumps, or Tet pumps, provide strong resistance to tetracyclines, whereas multidrug efflux systems export a broad spectrum of substrates, frequently including several drugs (Li and Nikaido, 2009; Grossman, 2016).

**Figure 4:**
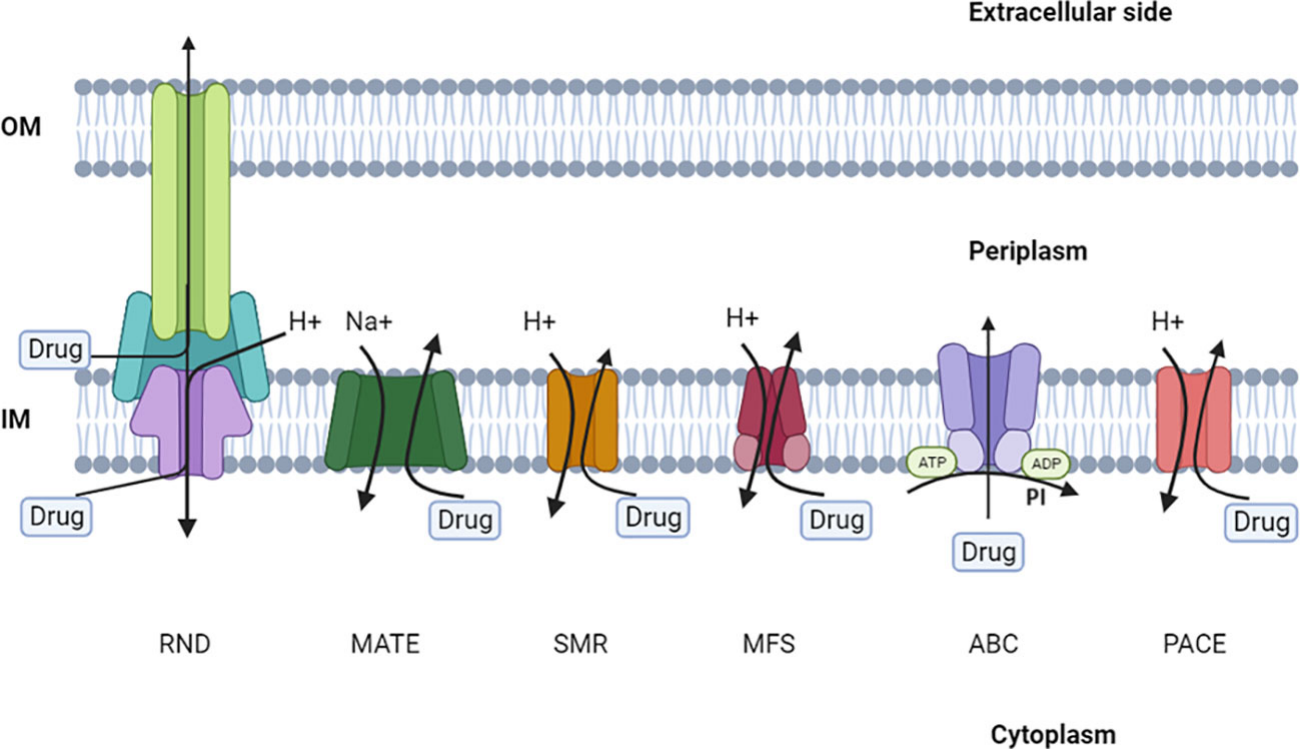
The main families of multidrug efflux pumps.

The seven unique efflux pump superfamilies that are present in bacteria have been rigorously described and characterized by microbiological, biochemical, and structural studies. Specific criteria, such as membrane topology, membrane-spanning properties, energy sources, substrate specialization, primary sequence similarity, and the stoichiometry of multi-subunit complexes, are used to categorize members of these families. Efflux pumps are classified into seven main categories: ATP-binding cassette (ABC), major facilitator (MFS), multidrug and toxic compound extrusion (MATE), small multidrug resistance (SMR), resistance-nodulation-cell division (RND), proteobacterial antimicrobial compound efflux (PACE), and p-aminobenzoyl-glutamate transporter (AbgT) (**Figure 4**) (Chetri et al., 2019; Nishino et al., 2021). It is worth mentioning that ABC transporters require ATP to function, while other efflux systems generate energy from the potential gradient of protons.

### 7.1 The ABC transporters

Usually, these transporters consist of four subunits: two ATP-binding domains and two membrane-spanning domains. The ABC transporters typically form homo- or heterodimers, each of which is made up of two half-transporters, when it comes to substrate export. One nucleotide-binding domain and one membrane-spanning domain make up each half-transporter. ABC pumps are different from other types of multidrug efflux pumps because they are categorized as primary transporters. This means that their transport function is directly connected to the hydrolysis of ATP, instead of relying on ion transport like other types of pumps (Baylay et al., 2019; Thomas and Tampé, 2020).

In this context, the lactococcal homodimeric ABC-type protein (LmrA) holds a distinctive position as the initial and most extensively examined MDR pump. LmrA is adept at transporting a diverse range of substances, including antimicrobial agents and chemotherapy drugs (Marshall and Bavro, 2019; Sharkey and O'Neill, 2019).

The flow of lipids like lipid A (endotoxin), which acts as the hydrophobic anchor for LPS, is mediated by two essential ABC transporters in *E. coli*, LmrA and MsbA. When compared to LmrA, MsbA has overlapping substrate specificities and confers multidrug resistance in *E. coli*. It’s interesting to note that MsbA deletion results in membrane disruption and cell death. This finding suggests that MsbA may be taken into account as a possible target of antibiotic treatment, particularly UPEC. Additionally, several ABC transporters, such as PatAB from *Streptococcus pneumoniae*, which moves fluoroquinolones, and MacAB from *E. coli*, which moves macrolides, impart antibiotic resistance (Padayatti et al., 2019).

### 7.2 The MacAB–TolC pump complex

The inner membrane transporter-outer membrane channel (MacAB-TolC) pump complex is home to an additional drug efflux mechanism of *E. coli* that has recently received attention. *E. coli* and other GNB, including pathogenic species, exhibit drug resistance and virulence characteristics that are influenced by the MacA-MacB-TolC assemble. These pumps control the transfer of outer membrane glycolipids, lipopeptides, protoporphyrin, and polypeptide virulence factors, such as the heat-stable enterotoxin II, in addition to the efflux of macrolide antibiotics (Fitzpatrick et al., 2017). The three main components of the MacAB-TolC efflux system are the periplasmatic membrane fusion protein (MPF), MacB, which recognizes drug molecules and activates efflux, and TolC, which allows antimicrobials to pass through the inner membrane. The MPF mediates interactions between the TolC and inner member transporter (Lu and Zgurskaya, 2013).

### 7.3 MFS efflux pumps

MFS pumps are the largest class of solute transporters and are primarily in charge of efflux-mediated resistance in Gram-positive bacteria, despite the fact that they are also present in GNB. They are made up of a single polypeptide chain that spans 12 or 14 membranes, and the substrate efflux is driven by the proton motive force. Less research has been done into MFS transporters that associate with TEPs. The proton-motive force uncoupler, carbonyl cyanide m-chlorophenyl-hydrazone (CCCP), and nalidixic acid are only a few examples of hydrophobic molecules that are resistant to EmrB and EmrA in *E. coli*. To identify MFS pumps associated with multidrug resistance more precisely, the Drug: H+ antiporters 1 (DHA1) and 2 (DHA2) can be employed, leveraging the insights from their crystal structures. YajR, EmrD, and MdfA in *E. coli* are examples of DHA1 transporters that have been specifically defined by their conformation. These transporters show the main helices that medicines preferentially target (Yin et al., 2006; De Gaetano et al., 2023).

Additionally, nothing is known about the MFS-based EmrAB-TolC tripartite efflux systems. Similar in length overall to AcrAB-TolC, the EmrAB-TolC complex may reveal how the adaptor protein joins TolC and EmrB entrenched in the inner membrane by a tip-to-tip connection between EmrA and TolC (Yousefian et al., 2021). More than ten years ago, the components of EmrAB-TolC were initially discovered in *E. coli*, where they conferred resistance to hydrophobic toxins like CCCP. Its overexpression results in greater resistance to hydrophobic proton uncouplers, nitroxoline, nalidixic acid, and thiolactomycin (Puértolas-Balint et al., 2020).

The multidrug transporter EmrD from the MFS is responsible for ejecting amphipathic substances over the inner membrane of *E. coli*. Detergents like sodium dodecyl sulfate and benzalkonium chloride can be transported by it. Through the efflux of arabinose, which encourages cell aggregation and biofilm formation, EmrD may play a crucial role in biofilm development (Koita and Rao, 2012; Alav et al., 2018). In addition, the 12-helix and 14-helix tetracycline transporters TetA(B) and TetA(K) from the bacteria *E. coli* and *S. aureus*, respectively, are classified as members of the MFS drug transporter family (Lynch, 2006).

### 7.4 SMR efflux pumps

SMR systems, or small multidrug resistance transporters, allow researchers to examine the essential conditions for active transport. They have four transmembrane helices and a little additional membrane domain, making them modest multidrug transporters. Small hydrophobic proteins with four transmembrane -helical spanners and roughly 100 amino acid residues make up the SMR family. SMR pumps encourage the solubilization of a variety of medications, including disinfectants, poisonous lipophilic compounds, or hazardous metabolites (Bay et al., 2008; Bay and Turner, 2009). Mobile drug resistance gene arrays usually contain the genes that encode SMR proteins (variously designated as *emrE*, *ynfA*, and *tehA*), which offer a broad selective advantage by providing resistance to ambient toxins that are widely present but only mildly harmful to microorganisms (Kermani et al., 2020). Small hydrophobic proteins with four transmembrane α-helical spanners and roughly 100 amino acid residues make up the SMR family (Hussein et al., 2022). Protein SMR for the GNB *E. coli*, EmrE is found in the inner membrane and offers resistance against a variety of antiseptic quaternary cationic compounds (QCCs). Tetracycline, tetraphenylphosphonium, ethidium bromide, and other toxic cationic hydrophobic substances, as well as various antiseptics and intercalating dyes, are only a few examples of the hazardous cationic hydrophobic substances that bacteria grow resistant to when EmrE is overexpressed (Hussein et al., 2022). The SMR gene family now includes the *E. coli* gene *ynfA*, which has also been found in Gram-positive and GNB species. It may be engaged independently, in conjunction with *tolC*, or in any other way through intricate control where the initially sensitive bacteria develop resistance. Two to six times more *ynfA* gene expression than *tolC* gene expression was observed (Sarkar et al., 2015). According to Ali et al., the three SMR pump genes (*emrE*, *ynfA*, and *tehA*) were distributed as follows: *emrE* 48 (96%), *ynfA* 50 (100%), and *tehA* 49 (98%) (Ali and Al-Dahmoshi, 2022).

MdfA, a membrane protein consisting of 410 amino acids, functions as a drug/proton antiporter. It imparts resistance to a range of lipophilic substances with cationic or zwitterionic properties, such as ethidium bromide, tetraphenylphosphonium, rhodamine, daunomycin, benzalkonium, rifampin, tetracycline, and puromycin. Surprisingly, MdfA also confers resistance to therapeutically significant antibiotics with unrelated chemical structures, including erythromycin, chloramphenicol, certain aminoglycosides, and fluoroquinolones (Edgar and Bibi, 1999).

When *mdfA* and *acrAB* are overexpressed together, quinolone resistance increases synergistically. The UPEC strain EC13049 was found to contain *mdfA*, which is responsible for resistance to aminoglycosides, phenicols, fluoroquinolones, tetracycline, rifamycin, and macrolides, according to a genome analysis (Zakaria et al., 2021).

Even in the absence of other particular genes, the expression of MdfA is said to provide extra resistance to clinically significant, chemically unrelated drugs (Zakaria et al., 2021). The rising resistance to levofloxacin was linked to the increased expression of the efflux pump-coding genes *acrA* and *mdfA*, according to research by Abdelhamid and Abozahra (2017) (Abdelhamid and Abozahra, 2017). This finding supports the idea that efflux pump systems play a role in fluoroquinolone resistance in urinary *E. coli* isolates. According to Morales et al., *mdfA* was the gene most frequently found in urine *E. coli* isolates. Ibrahim and Al-Zubaidi, 2023, demonstrated that 95% of bacterial isolates had both the *tolC* and *acrB* genes and that all bacterial isolates possess the gene (*mdfA*) (Ibrahim and Al-Zubaidi, 2023).

### 7.5 MATE efflux pumps

GNB frequently have MATE efflux pumps, or multidrug and toxic compound extrusion pumps. The size of MATE transporters is quite comparable to that of MFS transporters, and they generally consist of about (Hussein et al., 2022). Twelve α-helical transmembrane helices (TMHs) make up members of the MATE family, which use electrochemical ion gradients to induce the efflux of cationic and polyaromatic medicines. They use sodium antiport mechanisms or the proton motive force to generate power for efflux. When overexpressed, MdtK is a crucial MATE inner membrane transporter in *E. coli* that confers resistance to quinolone and fluoroquinolone (Eshaghi et al., 2021). Examples include the fluoroquinolones and benzalkonium chloride-transporting enzymes VcrM from *Vibrio cholerae*, MepA from *S. aureus*, and PmpM from *P. aeruginosa*. Fluoroquinolones are the main substrates that MATE transporters recognize. As shown in *Neisseria gonorrhoeae*, NorM is an example of a MATE efflux pump that shields pathogens from the harm caused by reactive oxygen species and exports antibiotics or antimicrobial cationic chemicals. Additionally, the MATE component DinF confers resistance to the antibiotic’s moxifloxacin, ciprofloxacin, and levofloxacin in pneumococci (Andersen et al., 2015).

### 7.6 PACE efflux pumps

PACE transporters are involved in the extrusion of biocides like chlorhexidine and are produced by highly conserved genes among bacterial species. SMR transporters and members of the PACE family of transporters have comparable secondary structures and sizes. The Acinetobacter chlorhexidine efflux protein I (or AceI) in *A. baumannii* is the PACE transporter that has been most thoroughly characterized. Recently, *P. aeruginosa* was shown to contain the novel PACE transporter PA2889. Regardless of pH, PA2880 transports chlorhexidine and produces dimers in a solution, similar to AceI (De Gaetano et al., 2023).

### 7.7 RND efflux pumps

The RND superfamily is thought to be the most significant group of efflux pumps since it grants MDR to a wide range of GNB species. The RND family of transporters is made up of three parts: an exterior membrane channel, an inner membrane pump, and a periplasmic adaptor protein that bridges the two channels (Alenazy, 2022). RND transporters take up substrates in the cytoplasm, periplasm, or the outer leaflet of the inner membrane. The inner-membrane protein (IMP), outer-membrane protein (OMP), and periplasmic adapter protein (PAP), which joins IMP and OMP, are the three proteins that make up the RND superfamily drug transporters. Numerous types of antibiotics, including fusidic acid, fluoroquinolones, tetracyclines, novobiocin, and chloramphenicol, can be extruded by RND pumps. One sort of substrate is an antibiotic, but there are also biocides, detergents, bile salts, metals, and things that the bacteria themselves produce, like virulence factors and iron-chelating siderophores (Munita and Arias, 2016).

The AcrAB-TolC drug efflux complex of *E. coli*, a member of the RND superfamily, is regarded as an archetypical drug efflux system in GNB and is likely the best-studied example. Other species, such as *Pseudomonas* spp.’s MexAB-OprM, MexCD-OprJ, and MexXY-OprM, have been shown to contain homologs of this system. *E. coli* that is resistant to carbapenem has been demonstrated to overexpress the efflux pumps AcrAB-TolC and AcrAD-TolC. According to one study, ertapenem resistance and AcrA overexpression are strongly correlated. Stress brought on by imipenem in *E. coli* also caused AcrB overexpression (Chetri et al., 2019). RND efflux pumps are typically chromosomally encoded, with plasmid-borne components being reported in only a small number of investigations. The gene cluster for the RND efflux pump, tmexCD1-toprJ1, was initially identified within *K. pneumoniae*. It was found in a conjugative plasmid of the IncFIA type, specifically in the plasmid pHNAH8I. This strain was isolated in 2017 from cloacal swab samples collected at a poultry farm in China (Lv et al., 2020).

The TMexCD-TOprJ determinant is a novel PMQR determinant that confers resistance to or reduced susceptibility to severalantibiotic classes, including quinolones, cephalosporins, phenicols, and tetracyclines aminoglycosides. Additionally, it can co-transfer between Enterobacteriaceae along with other mobile resistance genes as *mcr-8*. It was suggested that omeprazole resistance in UPEC is caused by mutations in the efflux repressor *acrR* and the *acrAB-TolC* genes (Chowdhury et al., 2019). Chetri et al. have discovered that there is a significant association between ertapenem and AcrA overexpression. Additionally, they noticed that when bacteria are exposed to imipenem, the AcrB system is overexpressed (Chetri et al., 2019).

Increased MICs of trimethoprim in *E. coli* have been associated with the AcrAB-TolC efflux system. It has been demonstrated that this system becomes more active due to a decrease in periplasmic glutathione levels, caused by loss-of-function mutations in the *gshA*, *grxA*, and *cydD* genes, which regulate glutathione content. This suggests that alterations in these genes may potentially be responsible for chromosomal resistance to trimethoprim. Overexpression of the levels of the AcrA-AcrB-TolC efflux pump complex, which includes QepA and OqxAB, leads to heightened resistance of UPEC bacteria against *β*-lactam antibiotics, with a particular impact on cefoxitin, as well as chloramphenicol, tetracyclines, and notably, quinolones (Ribeiro et al., 2020). According to Pantel et al., genes that display significant overexpression and encode efflux systems, which are less frequently observed in clinical resistance contexts (*acrEF*, *mdfA*, *yhiV*, *acrD*, and *tehA*), may potentially contribute to enhancing the efflux capacity of *E. coli* strains (Pantel et al., 2016). According to Tavio et al., the AcrA-AcrB-TolC efflux pump serves as the primary multidrug efflux transporter in *E. coli*, enabling the extrusion of fluoroquinolones and various other antimicrobial agents (Tavío et al., 2010). In another study conducted by Mottaghizadeh et al., no isolate had the *oqxA* or *oqxB* genes (Mottaghizadeh et al., 2020). Also, El-Mahdy et al, reported that neither OqxA nor OqxB was found in any of the *E. coli* samples. In 12 different *E. coli* isolates, *qepA* was found (El-Mahdy and Mashaly, 2022).

## 8 Membrane permeability mechanisms

Phospholipids, LPS, lipoproteins, and *β*-barrel porins make up the distinctive architecture of the OM of GNB. Toxic substances like bile acid and antibiotics are prevented from being transported by the OM, which acts as an extra barrier. As a result, substances with molecular weights more than 600 Da typically cannot pass through the GNB’s outer membrane. As a result, antibiotics like vancomycin and daptomycin, which have molecular weights more than 1400 Da, cannot cross the OM of GNB. Invasion, adherence, serum resistance, and antibiotic resistance all depend heavily on OMs. Some OMs in ESBL-producing bacteria have been removed, making it easier for resistance elements to transfer. The porins, which come in a variety of forms, are the OM proteins found in GNB in the highest abundance. Based on their action, they can be categorized as non-specific or specific porins (Choi and Lee, 2019).

In accordance with their functional structure, they are further divided into mono-, dimeric- or trimeric OM-spanning *β-*barrels porins. Porins play a pivotal role, not only in preserving the structural integrity of the GNB envelope, but also in facilitating the passive diffusion of various compounds. For example, the non-specific porin, known as outer membrane protein A (OmpA), enables the passive transport of a wide range of small molecules. This is a peptidoglycan-linked protein that engages with peptidoglycan through non-covalent interactions and possesses a flexible periplasmic domain. Porins are tightly linked to antibiotic resistance in GNB because they promote the passive diffusion of drugs across the OM (Choi and Lee, 2019). For instance, the non-specific porin OmpF has been shown to allow *β*-lactams and fluoroquinolones to enter the OM. As a result, some GNB, such as *E. coli, K. pneumoniae, Serratia marcescens, P. aeruginosa*, and *Enterobacter aerogenes*, were resistant to a number of *β*-lactam antibiotics. On the other hand, deletion of OmpA raised *A. baumannii’s* resistance to a number of antibiotics, including *β*-lactams. The complexity of porin-mediated antibiotic resistance, according to Choi et al., was demonstrated by their findings. Rather than transporting antibiotics, OmpA is critical for maintaining the integrity of the membrane. These OmpA functions require the C-terminal domain. OmpF is linked to membrane permeability and antibiotic transport, yet the *ompF* mutant displays antibiotic resistance characteristics. Increased susceptibility to antibiotic was not found. These effects of OmpF on membrane integrity could be the cause of these outcomes. A significant porin for the OM permeability for *β*-lactams is known to be OmpF. Multiple *β*-lactam antibiotics were much more resistant to the *ompF* mutant. A novel porin, YddB, which appears to be involved in the passive transport of novobiocin across the OM, was also discovered by them through their thorough analysis of porins peculiar to *E. coli*. The purpose of YddB, an outer membrane protein with a projected *β*-barrel shape, is unknown. Compared to the wild-type strain, the *yddB* mutant exhibits a markedly lower susceptibility to novobiocin. The deletion of any porin gene did not change the MIC of novobiocin, hence YddB appears to be a significant porin that is in charge of novobiocin’s OM penetration. The function of particular porins for antibiotic transport is only marginally significant, save from specific examples like YddB and LamB. Most non *β*-lactam antibiotics appear to penetrate the OM in a porin-independent manner, but the majority of *β*-lactam antibiotics are transported with non-specific porins, particularly OmpF and OmpC (Choi and Lee, 2019; Rodrigues et al., 2022).

The alteration of LPS by phosphoethanolamine (pEtN), galactosamine, hydroxylation, palmitoylation, and 4-amino-4-deoxy-L-arabinose (L-Ara4N) is linked to resistance to polymixins and cationic antimicrobial peptides. Among Enterobacteriaceae family species, the pmrHFIJKLM or pbgPE operon adds a 4-amino-4-deoxy-l-arabinose residue to lipid-A in the LPS structure. Due to this change, colistin’s affinity for LPS is reduced. Moreover, the pmrHFIJKLM operon is upregulated and the expression of the transmembrane protein (Mgr B) is downregulated by the PhoQ/PhoP and PmrAB signaling systems in response to acidic pH, cation concentration, and antimicrobial peptides. Clinical isolates of *E. coli* have been reported to show increased expression of the PhoQ/PhoP and PmrAB signaling systems due to point mutations or the insertion of sequences like IS5-like, IS1F-like, and ISKpn14 in the *mgrB* B gene. By using *mcr*-1-8 plasmids, another method of LPS modification is carried out. pEtN Transferase, which is encoded by Mcr-1 plasmids, changes the lipid in LPS molecules by including pEtN. Additionally, pEtN transfer is connected to the *pmrC* gene in Pmr signaling circuits. The CrrA/CrrB 2CS element and *crrC* appear to regulate PmrAB signaling circuits. There have been reports that the deletion or dysfunctional mutation of the *crrC* gene causes colistin resistance (Azam et al., 2021; Karami-Zarandi et al., 2023).

**Figure 5:**
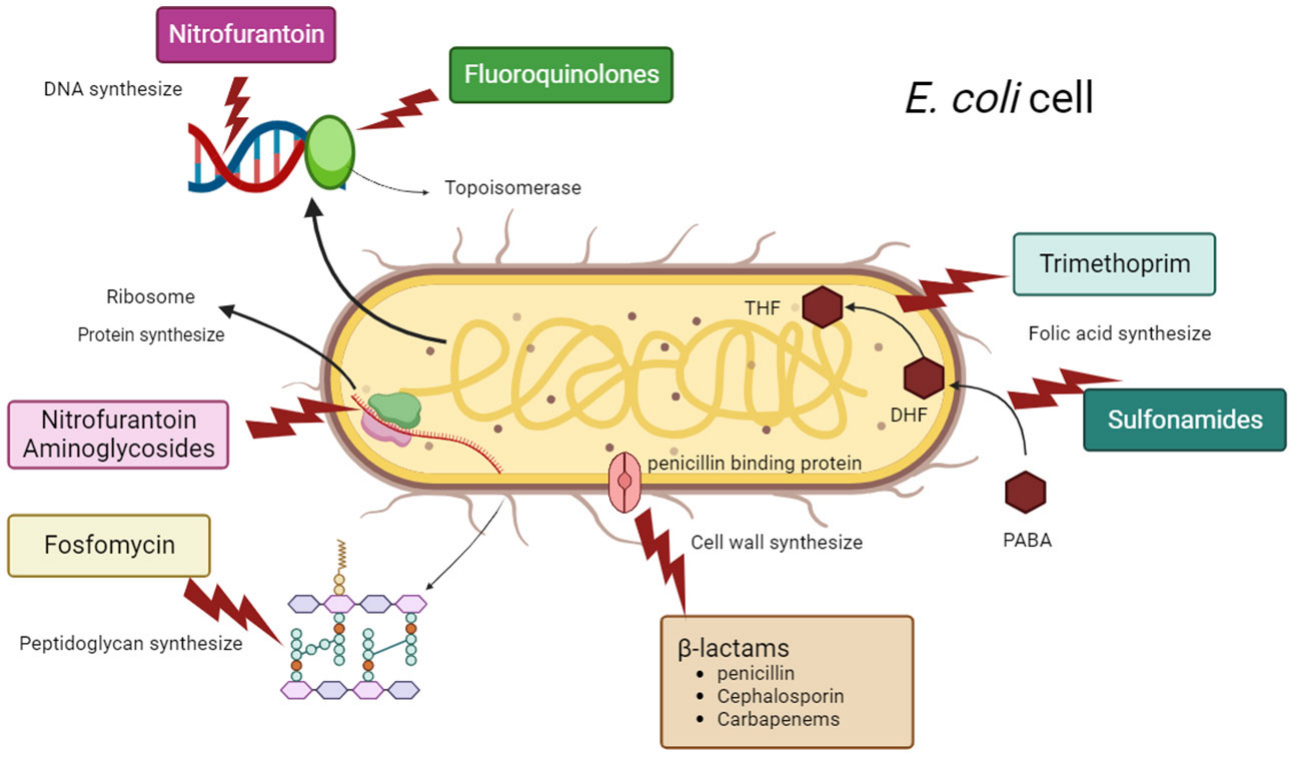
Antimicrobial therapy strategies and bacterial targets of antibiotics used to treat infections by UPEC.

Currently, antibiotics such as cefiderocol, colistin, fosfomycin, nitrofurantoin, and tigecycline can successfully treat UPEC strains that are resistant to carbapenem. Different nations have different levels of antimicrobial agent resistance (**Figure 5**) (Walker et al., 2022).

## 9 Cefiderocol

The FDA approved cefiderocol, a new type of cephalosporin antibiotic that acts as a siderophore, on November 14, 2019 for treating complicated UTIs in adults. Studies have demonstrated its effectiveness against carbapenem-resistant UPEC strains that belong to all Ambler classes, including those that produce ESBL, AmpC, KPC, and NDM-1 (El-Lababidi and Rizk, 2020; Walker et al., 2022).

Cefiderocol interacts with iron transport channels, allowing it to penetrate the bacterial outer membrane and infiltrate the periplasmic space, akin to a ‘Trojan horse’ strategy. Within this space, it achieves elevated concentrations, surpassing the resilience of most bacterial defense mechanisms, including efflux pumps, porins, and beta-lactamases. Subsequently, once inside, Cefiderocol binds to specific PBPs - PBP-3 and PBP-2, which are essential for cell wall synthesis. This binding inhibits the synthesis of peptidoglycan in the bacterial cell wall, ultimately resulting in cell lysis and bacterial death (Syed, 2021; Soriano et al., 2022). Cefiderocol can also circumvent other common *β*-lactam resistance pathways in GNB, like efflux pump up-regulation and porin deficiency (Bavaro et al., 2021).

Bassetti et al. conducted a phase 3 study involving patients with nosocomial pneumonia, bloodstream infections, or complicated UTIs caused by carbapenem-resistant GNB. The study found that cefiderocol was able to achieve microbiological eradication in 53% of patients with UTI, while only 20% of patients in the BAT group with UTI were able to achieve the same result (Bassetti et al., 2021). In a separate phase 2 trial involving patients with UTI caused by various carbapenem-resistant pathogens, including *E. coli*, cefiderocol was found to be more effective than imipenem-cilastatin, with a cure rate of 73% compared to 55% (p = 0.0004) (Portsmouth et al., 2018).Wang et al. reported A total of 26 *E. coli* isolates harboring NDM-5 showed high levels of cefiderocol resistance. They found 4 amino-acid insertions (YRIN/YRIK) at position 333 of PBP3 in the 26 *E. coli* isolates is associated with high levels of cefiderocol. According to Wang et al, 26 *E. coli* isolates that carried NDM-5 were found to be highly resistant to cefiderocol. The researchers identified four amino acid insertions (YRIN/YRIK) at position 333 of PBP3 in these isolates, which were linked to the high levels of cefiderocol resistance observed (Portsmouth et al., 2018).

## 10 Nitrofurantoin

Due to the limited rates of documented resistance, nitrofurantoin is still an effective first-line therapy and prevention for less severe UTIs (Tutone et al., 2022). The distinct and poorly known method of action of nitrofurantoin may explain why resistance to it develops more slowly than to other antimicrobials. The *nfsA* and *nfsB* genes produce enzymes called nitroreductases that are used by carbapenem-resistant GNB to break down nitrofurantoin into different antibacterial metabolites. These enzymes are not affected by the absence of oxygen (**Table 3**) (McOsker and Fitzpatrick, 1994). The lowering of NF and the consequent formation of hazardous chemicals are prevented by this mutation. To exert a bacteriostatic effect, the metabolites bind to bacterial ribosomes and stop protein synthesis in addition to inhibiting enzymes involved in the citric acid cycle and those in charge of DNA and RNA synthesis. For the first time, nitrofurantoin resistance in *E. coli* has been documented, and it has been determined that this resistance is caused by a mutation in the gene that codes for the oxygen-insensitive enzyme *nfsA*. The lowering of NF and the consequent formation of hazardous chemicals are prevented by this mutation (Mahdizade Ari et al., 2023). The *nfsA* and *nfsB* genes’ deletion and insertion mutations are the primary cause of nitrofurantoin resistance. Furthermore, resistance may result from mutations in the *ribE* gene, which codes for the enzyme lumazine synthase, which is necessary for the synthesis of the *nfsA* and *nfsB* cofactor flavin mononucleotide (Wan et al., 2021).

High levels of NF resistance (median MIC of 96 mg/ml) are mostly caused by mutations in the oxygen-insensitive nitroreductases *nfsA* and/or *nfsB* (Mahdizade Ari et al., 2023). Plasmid-mediated resistance to nitrofurantoin is another possibility. First off, a number of plasmid types carry the multidrug-resistant flux gene *oqxAB*, which is frequently flanked by IS26, which contains the transposon Tn6010 (Ho et al., 2016). High-level nitrofurantoin resistance is caused by *nfsA* inactivation together with expression of the *oqxAB* gene; however, it is not known if high level resistance can also be caused by *oqxAB* gene isolation. Unlike other types of resistant bacteria, those that are resistant to nitrofurantoin have been found to have a higher occurrence of efflux genes that also make them resistant to *β*-lactams, cephalosporins, aminoglycosides, carbapenems, and tetracyclines. Additionally, 51% of them have been shown to have XDR. According to reports, 1.1% and 1.8%, respectively, of *E. coli* bacteria identified from UTI patients in the United States and France are NF resistant (Whelan et al., 2023).

According to clinical studies, nitrofurantoin usage increased antibiotic resistance in comparison to trimethoprim and cotrimoxazole in the control group of patients who received this antibiotic as prophylaxis. The research by Fisher et al., in which patients had been on nitrofurantoin for 9–12 months before surgery, cited this as its most significant finding (Fisher et al., 2018). Additionally, these results support research by Pickard et al. that discovered higher antibiotic resistance in bacteria isolated from individuals who had taken oral nitrofurantoin as a preventative measure (Pickard et al., 2018). It is therefore advised that nitrofurantoin only be used to treat UTI cases where other antibiotic resistance is present (Mahdizade Ari et al., 2023).

## 11 Fosfomycin

A broad-spectrum phosphonic antibiotic with bactericidal action against both Gram-positive and Gram-negative pathogens, fosfomycin was initially developed in Spain in 1969 (Findlay et al., 2023). Both oral and intravenous preparations of fosfomycin are offered for usage. Fosfomycin can be taken orally as fosfomycin trometamol and fosfomycin calcium, whereas fosfomycin for intravenous use comes in a formulation that contains succinic acid as an excipient and disodium in a ratio of 1 g to 8 g. Although oral formulations have been demonstrated to help treat male UTIs and acute bacterial prostatitis, they are not indicated as a first-line treatment for uncomplicated UTIs in women with infections caused by *E. coli* (Whelan et al., 2023). This is because after injection, higher intraprostatic concentrations are also attained in addition to the urine. Similar to nitrofurantoin, fosfomycin’s resistance rates among UPEC are still low, maintaining its therapeutic efficacy in the treatment of UTIs. Chromosome- or plasmid-mediated mechanisms give fosfomycin resistance to bacteria. The majority of resistance is chromosomally mediated and prevents the antibiotic from reaching the bacterium (Gardiner et al., 2019). Additionally, the mechanism of action is distinct, making the emergence of co-resistance improbable. Nevertheless, opposition is growing, particularly among ESBL manufacturers (Shaikh et al., 2015). By binding to the cysteine 115 residue of the enzyme N-acetyl glucosamine transferase (MurA), which catalyzes peptidoglycan synthesis in *E. coli*, fosfomycin inhibits the first stage of peptidoglycan production (Borisova et al., 2014). Glycerol phosphate (GlpT) and hexose phosphate (UhpT), two transport mechanisms, are used by fosfomycin to penetrate the bacterial cell wall. Glycerol phosphate (GlpT) and hexose phosphate (UhpT), two transport mechanisms, are used by fosfomycin to penetrate the bacterial cell wall. Fosfomycin resistance can be brought on by amino acid changes in the active site of the MurA target or mutations in the chromosomal *glpT* and *uhpT* genes (Abdelraheem et al., 2023) (**Table 3**).

Another significant route for fosfomycin resistance is the generation of fosfomycin-inactivating enzymes (*fos* genes). Different fosfomycin-modifying enzymes, including FosA, FosB, FosC, and FosX, have been identified. These enzymes are encoded by the *fos* gene, which cleaves the oxirane ring to render fosfomycin inactive. FosA enzymes are the fosfomycin-modifying enzymes that are most commonly reported and are widespread in Enterobacteriaceae. The most often seen FosA determinant in *E. coli* is the FosA3 enzyme. Less often seen in *E. coli* are other *fos* genes including *fosA4*, *fosA5*, and *fosA6* (Arca et al., 1997; Loras et al., 2020). A significant level of resistance was recently conferred by an *E. coli* strain isolated from urine that encodes *fosA8*, a protein that shares 80% of its amino acid sequence with *fosA*. This gene was linked to the genome of the species Leclercia decarboxylata, which naturally resists fosfomycin and has a gene pool for transferrable resistance (Whelan et al., 2023). According to a study conducted in Mexico on 350 ESBL-producing *E. coli*, 60.5% of the fosfomycin-resistant strains were *fos*-producing, with the majority of these strains carrying *fosA3*. In 28.9% of the cases, resistance in the strains resulted from mutations within the antibiotic transport system (Galindo-Méndez et al., 2022).

Another plasmid-mediated resistance gene known as fosL1 was discovered in Swiss isolates and is thought to have been acquired by Tn7-related transposition. Other fosA genes and this gene had 57% to 63% amino acid similarity (Kieffer et al., 2020).

According to an agar dilution technique used in a recent investigation in Egypt, 37 (38.5%) of the 96 *E. coli* isolates were found to be fosfomycin resistant (MIC 256 g/ml). Four isolates carry two of the 21 *Fos* genes that were found in the isolates. In this investigation, the fosfomycin-resistant gene *FosA3* predominated, appearing in 11 isolates (47.8%), followed by the *FosA*, *FosC2*, *FosA4*, and *FosA5* genes, which were discovered in 8 (34.8%), 4, 17, 4, and 1 (4.3%) of the 23 isolates with positive phosphonoformate test results, respectively. None of the isolates had the *FosA6* or *FosB* genes (Abdelraheem et al., 2023).

A high incidence of antibacterial resistance was seen in the majority of the fosfomycin-resistant strains tested in this investigation, and 85.7% of these isolates were MDR. When compared to fosfomycin-susceptible isolates, cefazolin, cefoxitin, and ceftazidime resistance rates were considerably higher in fosfomycin-resistant isolates (P value 0.05) (Cheah et al., 2015; Loras et al., 2020).

Most frequently found on conjugative plasmids that also carry genes for ESBLs of the CTX-M subtype, *FosA3* is the most prevalent plasmid-mediated fosfomycin-resistant gene in *E. coli* isolates (Wachino et al., 2010; Sato et al., 2013; Cao et al., 2017). When solely ESBL-producing *E. coli* were taken into account, specific studies in Spain found that the rates of fosfomycin resistance among *E. coli* isolated from urine samples of adult patients were 2.7% and 3.8%, respectively (Loras et al., 2020). As a result of our findings, Abu El-Wafa and Abouwarda in Egypt discovered 24.1% fosfomycin resistance in UPEC isolated from patients of various ages (Mohamed Abu El-Wafa and Abouwarda, 2022). Out of 199 UPEC isolates tested in the UK, nitrofurantoin, the current first-line treatment for uncomplicated UTIs, has the lowest resistance rate both hospital and community settings (Carter et al., 2023).

## 12 Trimethoprim-sulfamethoxazole

Trimethoprim is frequently used with the sulphonamide medication sulfamethoxazole, which inhibits the production of dihydrofolic acid, a cofactor necessary for the efficient synthesis of bacterial DNA (Masters et al., 2003). Mobile genetic elements, plasmids, and integrons are strongly related to resistance to trimethoprim and sulfamethoxazole (Stephens et al., 2020; Mahmood and Ibrahim, 2023). The chromosomal folA and folP of a species that naturally resists trimethoprim and sulphonamides are the origin of the genes that encode these resistance proteins (Sánchez-Osuna et al., 2020). Several genes in the Dfr (di-hydrofolate reductase) family give resistance to trimethoprim. There are more than 30 *dhfr* genes; *dfrA1* and *dfrA17* are the two most prevalent in UPEC (Al-Assil et al., 2013). These *dfrA* genes are frequently located within integrons and on plasmids near *sul* genes, which provide resistance to sulfamethoxazole. They are also frequently discovered in bigger resistance islands, which frequently include the virulence genes *fimH*, *fyuA*, irp2, and *sitA* as well as the chromate-resistance gene chrA, the aminoglycoside genes *aac(3)-IIa*, *aac(6)-Ib-cr*, and *aadA2*, and the gene *bla_CTX-M_*. Trimethoprim-sulfamethoxazole resistance has expanded widely among UPEC due to the intrinsic mobility linked with these resistance determinants (Zeng et al., 2021; Rahman et al., 2022; Mahmood and Ibrahim, 2023). Prior prescriptions of nitrofurantoin were discovered to be related with a decreased frequency of trimethoprim resistance. Selective pressure offered by earlier antibiotic therapy, notably the use of extended-spectrum penicillin, has been proven to cause trimethoprim resistance (Mulder et al., 2019).

Even though these mobile determinants have been proven to have the most impact on resistance to these medications, chromosomal mutations and efflux have also been shown to contribute to resistance to trimethoprim-sulfamethoxazole. In terms of efflux, research indicates that loss-of-function mutations in the GSH content-regulating genes *gshA, grxA*, and *cydD* lead to decreased periplasmic glutathione concentration, which in turn enhances the AcrAB-TolC efflux pathway (Song et al., 2021). This raises the possibility that changes in these genes might be the cause of chromosomal resistance to trimethoprim. Additionally, research that examined an *E. coli* knockout library discovered that the deletion of *mgrB* led to trimethoprim resistance. Significantly elevated genes in *mgrB*-deleted strains included *phoP*, *phoQ*, and *folA*. This is due to the fact that *mgrB* controls the expression of *folA*, demonstrating the possibility of trimethoprim resistance in several genes related to folate metabolism (Shi et al., 2021) (**Table 3**).

**Table 3:** Antibiotic resistance in *E. coli* is commonly caused by various mechanisms such as chromosomal mutations, enzymatic activities, and efflux pumps which areshown in **Table 3**.

**Nitrofurantoin**	Chromosomal Mutations	*nfsA, nfsB, ribE*
	Efflux Mediated (Plasmid Encoded)	*oqxAB*
**Fosfomycin**	Chromosomal Mutations	*murA, glpT, uhpT, uhpA, ptsI* and *cyaA*
	Enzymatic Mechanism (Plasmid Encoded)	*fosA),fosA3*, *fosA4*, *fosA5*, and *fosA6*(*fosL, fosB*, *fosC* (*fosC2*), and *fosX*
**Trimethoprim**	Chromosomal Mutations	*mgrB*
	Enzymatic Mechanism (Plasmid Encoded)	*dfrA1, dfrA5, dfrA7, dfrA12, dfrA14*, *dfrA16, dfrA17, dfrA19, dfrA8, dfrA14, dfr2d, dfrA3, dfrA9, dfrA10, dfrA24, dfrA26, dfrA27, dfrA32, drfb4*
	Efflux Mediated	*acrAB-tolC*
**Sulfamethoxazole**	Chromosomal Mutations	
	Enzymatic Mechanism (Plasmid Encoded)	*sul1, sul2, sul3*
	Efflux Mediated	
**Fluoroquinolones**	Chromosomal Mutations	*gryA, gyrB, parC*
	Enzymatic Mechanism (Plasmid Encoded)	*qnrA, qnrB, qnrC, qnrS, qnrD, qnrE, qnrVC, aac(60)-Ib-cr*
	Efflux Mediated	*acrB*, *qepA*, *oqxAB*
**Colistin**	Chromosomal Mutations	*PhoPQ, PmrAB/BasRS, mgrB*
	Enzymatic Mechanism (Plasmid Encoded)	*mcr-1–9*
	Efflux Mediated	*marAB/soxS* upregulation *marR* mutant

## 13 Colistin resistance

According to recent data, a significant percentage of infections caused by CRE in the USA required the use of colistin and polymyxin B, which are considered last-resort drugs. This is often due to the fact that the bacteria have developed resistance to more commonly used antibiotics. Colistin is particularly effective against GNB, including Enterobacteriaceae and other types such as *P. aeruginosa* and *A. baumannii*. It was first found in 1947 (Karami-Zarandi et al., 2023b). The medicine was initially prescribed frequently, but as a result of its ineffectiveness and numerous side effects, including nephrotoxicity and neurotoxicity, its use gradually decreased. Despite these drawbacks, the WHO regards colistin as a “highest priority critically important antimicrobial for human medicine” due to its capacity to treat infections brought on by bacteria that are ordinarily resistant to antibiotic treatment. In Enterobacteriaceae, chromosome point mutations and changes, particularly in genes encoding two-component regulatory systems, such as PhoPQ and PmrAB/BasRS, can result in the acquisition of colistin resistance in *E. coli* and other MDR pathogens like *K. pneumoniae* or *E. cloacae* (Bialvaei and Samadi Kafil, 2015) (**Table 3**).

Because of these mutations, eptA and the arnBCADTEF operon are expressed constitutively, which in turn causes lipid A to acquire 4-amino-4-deoxy-l-arabinose (l-Ara4n) and/or pEtN groups, modifying LPS. In 2016 saw the discovery of plasmid-encoded colistin resistance in a number of isolates from both humans and livestock in addition to chromosomal alterations. A single gene called mobilized colistin resistance-1 (*mcr-1*) produces a transferase enzyme that alters the bacterial lipid-A lipopolysaccharide. Additionally, this gene makes *E. coli* resistant to polymyxin drugs. The fact that *mcr* is a transposable genetic element and has been identified on a variety of bacterial plasmids, including the IncX4 and IncHI2, makes it possible for horizontal gene transfer. Since then, research on enterobacteria with *mcr* genes has been reported from all over the world, and as of now, variations with numbers ranging from *mcr-1* to *mcr-10* have been described (Bastidas-Caldes et al., 2022). There have been reports of 10 different classes of *mcr*, all of which encode pEtN transferases. Subsequent research showed that *mcr-1* is distributed globally in a variety of different Enterobacteriaceae, but mainly *E. coli* (Anyanwu et al., 2023).

As a result, LPS changed with pEtN is responsible for MCR-mediated colistin resistance, whereas LPS modified with l-Ara4n and/or pEtN alterations is responsible for resistance conferred by chromosomal mutations. LPS modification takes place in the outer leaflet of the CM in both colistin resistance caused by chromosomal mutations and MCR (Humphrey et al., 2021).

Although not all LPS molecules are transformed in either membrane, this causes modified LPS to be present in both the CM and OM. Both l-Ara4n and pEtN have positive charges, which lowers lipid A’s anionic charge and is hypothesized to lower the lipid’s affinity for colistin’s cationic peptide ring. The presence of unaltered LPS molecules, which the polymyxin antibiotic can bind to, there is evidence that colistin can still harm the OM of MCR-1-producing *E. coli*. The fact that colistin did not permeabilize the CM of an MCR-1-producing strain, despite harming the outer membrane of resistant bacteria, explains why the bacteria are resistant to death or lysis and can withstand exposure to polymyxin. The MCR-1 strain is protected against colistin but not the outer membrane due to the high concentration of modified LPS and low total abundance of LPS in the cell membrane. This is because there are fewer unmodified LPS molecules in the cell membrane that colistin may target (El-Sayed Ahmed et al., 2020; Humphrey et al., 2021).

According to Humphery and colleagues, they found out that MCR-1-mediated resistance to colistin can shield *E. coli* from damage caused by CM through the modification of LPS with pEtN. However, this resistance does not prevent OM permeabilization. Although all MCRs are pEtN transferases, the researchers also noticed that the different *mcr* gene families have varying abilities to support *E. coli’s* survival and growth when exposed to colistin. Nonetheless, MCR-1 has consistently been found to be the most effective in protecting colistin due to the limited extent of CM permeabilization (Humphrey et al., 2021).

Colistin works by attaching itself to lipids through a binding process. a fraction of LPS found in GNB’s outer membrane, like that of *E. coli*. In particular, colistin binds to LPS molecules in the cytoplasmic membrane’s outer leaflet, rupturing it and killing the bacteria. The phosphate groups on the lipid A subset of LPS that are negatively charged enhance this interaction (El-Sayed Ahmed et al., 2020).

It is suggested that the primary cause of cell death is the ensuing breakdown of the inner membrane. Previous research in *E. coli* has shown that mutations in the sensor histidine kinase PmrB are a significant cause of colistin resistance. These mutations result in the constitutive production of the enzymes ArnT and EptA, which add positive charges to the phosphate groups of lipid A (4-amino-4-deoxy-L-arabinose and pEtN, respectively), reducing the affinity of colistin for lipid A. The acquisition of mobile genetic elements carrying *mcr* genes, which also results in the decorating of lipid A with pEtN, offers a different route toward colistin resistance (Janssen and van Schaik, 2021). According to Knopp et al., novel auxiliary peptides can also result in colistin resistance by interacting directly with PmrB and activating the two-component system. MCR stands for mobilized colistin resistance; LPS for lipopolysaccharide, and the first mcr gene was discovered in 2016 (Knopp et al., 2021). The distribution of *mcr* genes in *E. coli* differs significantly globally, according to a systematic review by Bastidas-Caldes et al. The overall crude prevalence was 6.52% (n = 11,583/177,720), with a non-clinical isolate prevalence of 8.71% (n = 15,001/172,140) and a clinical isolate prevalence of 1.76% (n = 1020/58,033) (Bastidas-Caldes et al., 2022).

## 14 Conclusion

After decades of antibiotic use, *E. coli* went from being a harmless bacterium to once again posing a hazard to human health. MDR strains of *E. coli* were created by this bacteria’s innate capacity to absorb genetic material from other bacterial species at the dawn of the antibiotic era. As more antibiotics were used, the primary resistant strains continued to be under selective pressure, and new resistant strains were chosen that had acquired antibiotic resistance by horizontal gene transfer or spontaneous mutation. This bacteria’s metabolism was modified in special clones to promote efficient energy use and better replication. Clones with a wide range of antibiotic resistance and an effective metabolism spread around the globe. Even though these clones are extremely resistant to practically all antibiotics, recent research suggests that adequate stewardship programs could improve the efficacy of the antibiotics now in use. For instance, the prevalence of ESBL-producing bacteria will decline if third-generation cephalosporins are in some cases replaced with PIP/TZM. Additionally, colistin can be used as part of a therapy against pathogenic germs when combined with other medications. A significant factor in the creation and spread of colistin resistance among GNB is the drug’s unprecedented use in human medicine, animal husbandry, aquaculture, and agriculture. Understanding the complexity of antibiotic resistance mechanisms in *E. coli* is crucial for the development of effective strategies to combat this growing public health crisis. By reviewing these mechanisms, we hope this will increase opportunities for the design of innovative therapeutic approaches and the implementation of prudent antibiotic stewardship practices to preserve the efficacy of current antibiotics and ensure a sustainable future for healthcare. However, there is an urgent need for more research into the resistance mechanisms in *E. coli.*

## Author contributions

SN: Investigation, Validation, Writing – original draft, Writing – review & editing. JG: Investigation, Validation, Writing – original draft, Writing – review & editing. MH: Conceptualization, Investigation, Validation, Writing – original draft, Writing – review & editing.

## Glossary

**Table:**

UPEC	Uropathogenic *E. coli;* UTIs, urinary tract infections
ESBLs	extended-spectrum *β*-lactamases
HUS	Hemolytic uremic syndrome
AN	amikacin
CE	cephalosporins
CAZ	ceftazidime
CFP	cefoperazone
FEP	cefepime
PEF	piperacillin
PTZ	piperacillin-tazobactam
CP	ciprofloxacin
GM	gentamicin
MEM	meropenem
INN	netilmicin
CFX	cefoxitin
CTX	ceftriaxone
CFZ	cefazolin
ETEC	Enterotoxigenic *E. coli*
EPEC	enteropathogenic *E. coli*
EHEC	enterohemorrhagic *E. coli*
EIEC	enteroinvasive *E. coli*
EAEC	enteroaggregative *E. coli*
DAEC	diffusely adherent *E. coli*
NMEC	neonatal meningitis *E. coli*
MDR	multidrug-resistant
XDR	extremely drug-resistant
CREC	carbapenem-resistant *E. coli* isolates
MBL	metallo-*β*-lactamases
BLBLIs	The *β*-lactam *β*-lactamase inhibitor combinations
A/S	ampicillin/sulbactam
A/C	amoxicillin/clavulanic acid
PTZ	piperacillin/tazobactam
CZA	ceftazidime/avibactam
C/T	ceftolozane/tazobactam
PBP	penicillin binding proteins
cIAI	complex intra-abdominal infections
BL-BLI	broad-spectrum *β*-lactam-*β*-lactamase inhibitor
KPC	*K. pneumoniae* carbapenemase
CDC	Centers for Disease Control and Prevention
MVB	Meropenem/vaborbactam
VABP	ventilator-associated bacterial pneumonia
HABP	pneumonia acquired in a hospital setting
IMI-REL	imipenem-relebactam
AMEs	aminoglycoside-modifying enzymes
ANTs	aminoglycoside nucleotidyltransferases or adenylyltransferases
APHs	aminoglycoside phosphotransferases
AACs	aminoglycoside acetyltransferases
topo IV	topoisomerase IV
PMQR	quinolone resistance mediated by plasmids
QRDR	quinolone resistance-determining region
ABC	ATP-binding cassette
MFS	major facilitator
MATE	multidrug and toxic compound extrusion
SMR	small multidrug resistance
RND	resistance-nodulation-cell division
PACE	proteobacterial antimicrobial compound efflux
AbgT	p-aminobenzoyl-glutamate transporter
LmrA	the lactococcal homodimeric ABC-type protein
MPF	membrane fusion protein
AcrB or MacB	inner membrane transporter
TolC	outer membrane channel
MacAB-TolC	inner membrane transporter-outer membrane channel
CCCP	carbonyl cyanide m-chlorophenyl-hydrazone
QCCs	quaternary cationic compounds
TMHs	transmembrane helices
IMP	inner-membrane protein
OMP	outer-membrane protein
PAP	periplasmic adapter protein
OmpA	outer membrane protein A
pEtN	phosphoethanolamine
MurA	enzyme N-acetyl glucosamine transferase
GlpT	Glycerol phosphate
UhpT	hexose phosphate
fos genes	osfomycin-inactivating enzymes.
